# Sex differences in gastrointestinal cancers incidence and mortality among adolescents and young adults in China: an age-period-birth cohort study

**DOI:** 10.1515/jtim-2026-0028

**Published:** 2026-08-12

**Authors:** Feilong Ning, Chen Huang, Sihan Zhang, Sichen ZhuLi, Zhiping Yang, Chundong Zhang, Xiaoliang Gao, Daiming Fan

**Affiliations:** State Key Laboratory of Holistic Integrative Management of Gastrointestinal Cancers and National Clinical Research Center for Digestive Diseases, Xijing Hospital of Digestive Diseases, Fourth Military Medical University, Xi'an, Shaanxi Province, China; Department of Plastic and Reconstructive Surgery, Xijing Hospital, Fourth Military Medical University, Xi'an, Shaanxi Province, China; School of Basic Medicine, Fourth Military Medical University, Xi'an, Shaanxi Province, China; Department of Surgical Oncology and Central Laboratory, The Fourth Affiliated Hospital of China Medical University, Shenyang, Liaoning Province, China

**Keywords:** age-period-cohort analysis, China, early-onset, gastrointestinal cancer

## Abstract

**Background and Objectives:**

Early-onset gastrointestinal cancers in adolescents and young adults (< 50 years) from a unique subgroup with unclear epidemiological features. We aimed to estimate the burden of early-onset gastrointestinal cancers in China, focusing on the associations with age, period, and birth cohort effects.

**Methods:**

Cancer-specific estimates for six major types of early-onset gastrointestinal cancers in China were derived from Global Burden of Disease (GBD) 2021. We analyzed the incidence and mortality, and calculated temporal trends, mortality-to-incidence ratio, and age distributions. We used an age-period-cohort analysis to estimate longitudinal age trends (age effects) and period/birth cohort relative risks (period/birth cohort effects) during 1990–2021, and calculated overall annual percentage changes (net drifts).

**Results:**

There were 6.52 million incident cases and 4.32 million deaths due to early-onset gastrointestinal cancers in China during 1990–2021. Net drifts in incidence increased for colorectal (1.17% to 1.77%) and Gallbladder cancer (1.16% to 1.81%), while decreases were observed for esophageal cancer (-3.37% to -2.58%), gastric (-1.86% to -1.14%) and liver cancer (-1.75% to -1.06%) during 1990–2021. Period effects on incidence risk decreased for esophageal, gastric, and liver cancers from 1997 to 2021, but increased for colorectal and gallbladder cancers from 1997 to 2021. Birth cohort effects on incidence risk decreased for esophageal cancer (1945–1999), gastric (1945–2004) and liver cancer (1965–2004), whereas increases were seen for colorectal (1945–2004) and gallbladder (1945–2004). For pancreatic cancer, however, birth cohort effects remained largely stable across all cohorts. The age, period, and birth cohort effects on incidence and mortality were similarly consistent.

**Conclusions:**

These results provided new insights into the temporal trends in early-onset gastrointestinal cancers and their associations with age, period, and birth cohort effects, and may help to guide future public health policies for cancer control in China. The distinct temporal trends and sex-specific disparities identified in this study underscore the need for differentiated prevention and early screening strategies for gastrointestinal cancers among young adults in China.

## Introduction

Gastrointestinal cancer is a leading contributor to the global cancer burden, accounting for over a quarter of all cancer cases and a third of cancer-related mortality worldwide.^[1, 2]^ China has the highest cancer burden in the world, including about 40% of all new cases and 44% of all deaths due to gastrointestinal cancers.^[3]^ The incidence of gastrointestinal cancers has declined globally;^[4, 5, 6, 7]^ however, the incidence of early-onset (*i.e*., < 50 years) cases has been rising steadily in many countries.^[8, 9, 10, 11, 12]^ Notably, the incidence of early-onset gastrointestinal cancers, *i.e*., esophageal, gastric, colorectal, liver, gallbladder and biliary tract, and pancreatic cancers, is predicted to increase worldwide by 14.6% to 16.8% from 2020 to 2040.^[13]^ This urgent situation has underscored the need to accurately quantify the burdens and temporal trends of early-onset gastrointestinal cancers, especially in China, which accounts for approximately 20% of the global population. However, it remains a significant challenge to fully understand the epidemiological features of early-onset gastrointestinal cancers in China, which differ notably from later-onset cancers (*i.e*., ≥ 50 years).

Adolescents and young adults aged 15–49 years represent a diverse population experiencing various physical, emotional, and psychosocial changes as they navigate the transition into adulthood. Early-onset gastrointestinal cancers in this age group comprise a distinct subgroup with unique epidemiology. However, their features are not fully understood. The definition of the age range for early-onset cancer varies, especially the upper age limit, and we therefore used a definition of onset at < 50 years, as the widest age range definition of early-onset cancer, to allow comparisons with other studies in this population.^[8,10,12,14]^ The reasons underlying the global increase in early-onset gastrointestinal cancers are still poorly understood; however, it is crucial to understand the epidemiological trends of early-onset cancer as a sentinel for the future cancer burden in older adults, in whom most cancers will occur.^[11]^ Notably, a better understanding of the changing epidemiological trends of early-onset cancers will provide important information for their effective prevention, early detection (*i.e*., age for first routine screening), quality cancer care, resource allocation, and strategy adjustment, especially in China, which has been making efforts to confront its rapidly increasing cancer burden.^[15]^ Furthermore, it remains unclear if the incidence and mortality risk of early-onset gastrointestinal cancers in China differ according to physiological age, time period, or birth cohort. There is thus an urgent need for in-depth analyses of the up-to-date temporal trends and age, period, and birth cohort effects on the risks of early-onset gastrointestinal cancers in China.

To address this urgent problem, we analyzed data for six major types of early-onset gastrointestinal cancers in China using data derived from the Global Burden of Disease 2021 study (GBD 2021), with the aim of providing up-to-date information on epidemiological trends from 1990 to 2021. Here, we analyzed the incidence and mortality for the six types of cancer and calculated the temporal trends, male-to-female ratio, mortality-to-incidence ratio, and age distributions. We also used an age-period-cohort analysis to estimate the annual percentage changes (local drifts) for each age group and the overall annual percentage changes (net drifts) for the whole population. Furthermore, we calculated the fitted longitudinal age trend (age effects) and period and birth cohort relative risks (period and birth cohort effects) during 1990–2021.

## Methods

### Data source

We obtained data from the GBD 2021 (http://ghdx.healthdata.org/gbd-results-tool). Details of the methods and processes used in GBD 2021 for quantifying incidence and mortality have been described elsewhere.^[16, 17]^ This study did not need approval because it used publicly available data, and was reported according to the Strengthening the STROCSS guidelines.^[18]^

### Definitions of early-onset gastrointestinal cancers

We included six major types of early-onset gastrointestinal cancers in China, including esophageal, gastric, colorectal, liver, gallbladder, and pancreatic cancer. “Gallbladder cancer” throughout this study refers to “gallbladder and biliary tract cancers”. Notably, we applied an age definition of < 50 years, as the most-encompassing age range definition of early-onset cancer, allowing for comparability with other studies in this population;^[8,10,12,14]^ however, we acknowledge the limitations of applying this cutoff value. We extracted data on the incidence and mortality for this age range overall (< 50 years) and for the following age subgroups: 15–19, 20–24, 25–29, 30–34, 35–39, 40–44, and 45–49 years. The six major types of gastrointestinal cancers were defined based on the International Classification of Diseases, version 10 (ICD-10) (Figure 1).

**Figure 1:**
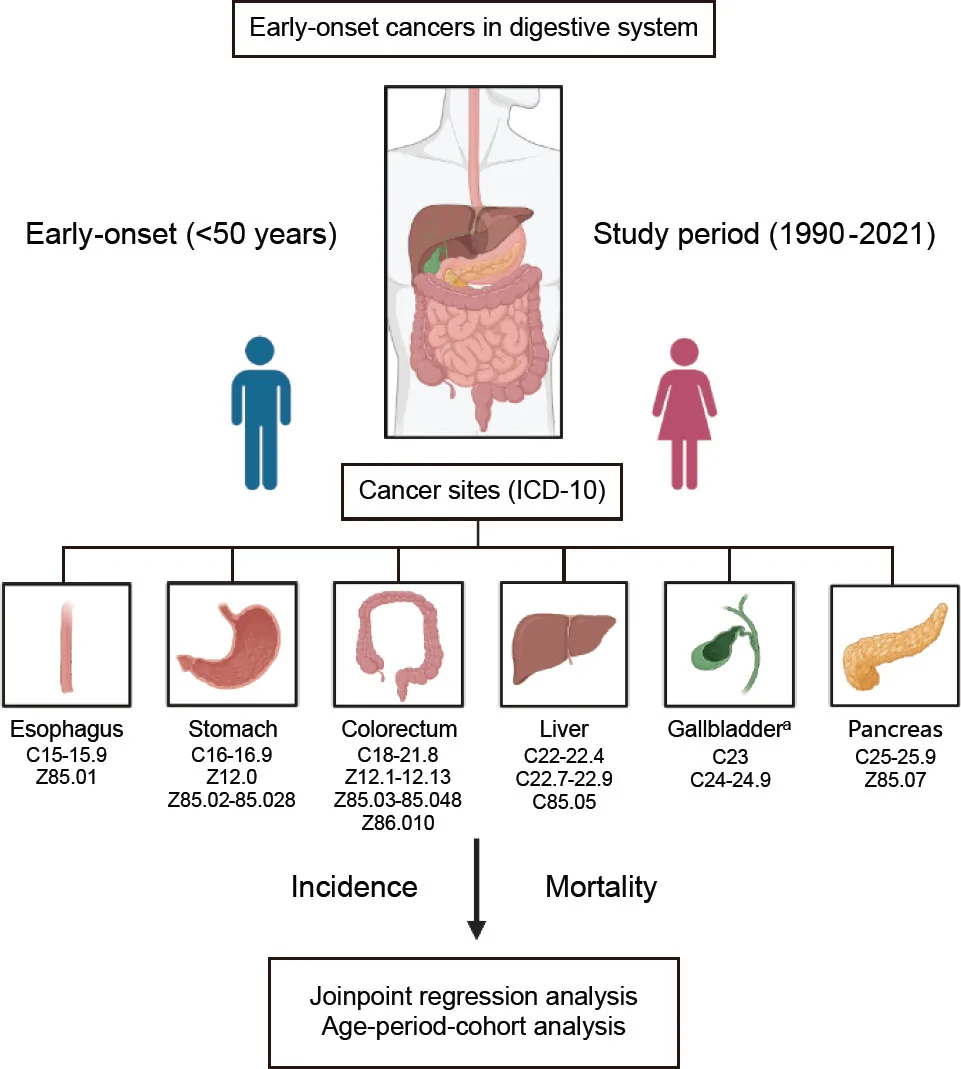
Flow chart for the inclusion of six major types of early-onset gastrointestinal cancers according to ICD-10. *Gallbladder and biliary tract cancers. ICD-10: International Classification of Diseases, 10th revision.

### Statistical analysis

Incidence and mortality rates were reported per 100,000 person-years, with 95% uncertainty intervals (UIs). The male-to-female ratio was calculated as the number of new male cases (or deaths) divided by the number of new female cases (or deaths). The mortality-to-incidence ratio was calculated as the number of deaths divided by the number of incident cases.^[19]^ We calculated the age distributions of the relative incidence and number of deaths in each age group. We estimated the average annual percent changes in incidence and mortality using Joinpoint regression. We also used an age-period-cohort analysis to estimate the annual percentage changes (local drifts) for each age group and the overall annual percentage changes (net drifts) for the whole population, and calculated the fitted longitudinal age trend (age effects) and period and birth cohort relative risks (period and birth cohort effects) during 1990–2021. For the age-period-cohort analysis, the model was fitted using the point estimates of incidence and mortality rates from GBD 2021. The 95% confidence intervals (CIs) for all age-period-cohort parameters were derived from the model itself and reflect the uncertainty in the age-period-cohort estimates, and thus do not propagate the posterior uncertainty distributions from the GBD 2021 data source. The association between Socio-demographic Index (SDI) and incidence/mortality rates was assessed using Gaussian process regression based on global, regional, and national data from 1990 to 2021. All statistical tests were two-sided, with a *P* value < 0.05 indicating statistical significance.

### Joinpoint regression analysis, 1990–2021

To comprehensively evaluate long-term temporal trends, we conducted joinpoint regression analysis for the entire study period (1990–2021), and because it is crucial to understand the recent temporal trends in incidence and mortality rates, we therefore estimated the annual percentage change (EAPC, % per year) with 95% confidence intervals (95% CI) from 2010 to 2021.^[20]^ Unlike simple linear regression, which assumes a constant trend over the entire study period, the joinpoint model identifies points in time (joinpoints) where the trend changes significantly. The incidence or mortality rate was considered to show an upward trend if the EAPCs and 95% CI were > 0, a downward trend if both were < 0; and to be stable if the 95% CI of EAPC included 0, as described elsewhere.^[21]^ The analyses were conducted using Joinpoint software, version 4.7.0.0 (National Cancer Institute, Rockville, MD, USA).

### Age-period-cohort analysis, 1990–2021

Age-period-cohort analysis is crucial for assessing the temporal trends in population-based disease rates, *i.e*., cancers.^[22, 23]^ The Age-Period-Cohort analysis was employed to disentangle the complex temporal trends in early-onset gastrointestinal cancer rates into three distinct components: age, period, and birth cohort effects. This approach is particularly suited for our study because it allows us to determine whether the observed changes in incidence and mortality are driven by factors associated with physiological aging (age effect), changes in diagnostic techniques, screening policies, or environmental exposures affecting all age groups simultaneously at a given calendar time (period effect), or by risk factors specific to generations born in different eras (birth cohort effect). The use of the age-period-cohort analysis is crucial for generating hypotheses about the etiological factors behind the observed trends, which cannot be achieved with simpler trend analyses.^[23]^ A web tool for age-period-cohort analysis designed by the US National Cancer Institute (http://analysistools.nci.nih.gov/apc/) has been widely used to estimate underlying trends in incidence and mortality rates, and the associations with age, period, and birth cohort effects.^[24]^ In this study, the analysis was performed using this tool, which implements the intrinsic estimator method. This method addresses the fundamental identifiability problem in age-period-cohort analysis through a normalization constraint that sets the geometric mean of the period and cohort coefficients to 1, thereby solving the well-known identification problem and providing robust, interpretable estimates. In this model, local drift indicated the annual percentage change in incidence or mortality (% per year) for each age group (from 15–19 to 45–49 years), and net drift indicated the annual percentage change in incidence or mortality (% per year) for the whole population (15–49 years). Furthermore, the model calculated fitted longitudinal age trend (age effects), period relative risks (period effects), and cohort relative risks (birth cohort effects).^[24]^ Since National Cancer Institute age-period-cohort web tool does not provide formal goodness-of-fit statistics (*e.g*., deviance, Akaike Information Criterion), the adequacy of the model fit was assessed by visually inspecting the agreement between the observed and fitted rates, consistent with the tool's standard practice.

In the age-period-cohort analysis, all age and period groups must have equal intervals (range, 1–10 years); for instance, if 5-year age groups are selected, then 5-year period groups must also be applied.^[24, 25]^ Given that the data for 5-year age groups is available from the GBD 2021, we therefore calculated incidence, mortality, and populations in consecutive 5-year-periods. The fitted longitudinal age trend in incidence or mortality rate (age effects) applied successive 5-year age groups from 15–19 to 45–49 years for gastric, colorectal, liver, and pancreatic cancers, and from 20–24 to 45–49 years for esophageal and gallbladder cancers, due to the lack of data for 15–19 year olds. The period relative risks for incidence and mortality rate (period effects) were calculated as the ratios of age-specific rates in each period group. The birth cohort relative risks of incidence and mortality rate (birth cohort effects) were calculated as the ratios of the age-specific rates in 12 consecutive birth cohorts for gastric, colorectal, liver, and pancreatic cancers, including those born from 1945–1949 (median, 1947) to 2000–2004 (median, 2002) and in 11 consecutive birth cohorts for esophageal and gallbladder cancers, including 1945–1949 (median, 1947) to 1995–1999 (median, 1997), with 1960–1964 (median, 1962) as the reference birth cohort. The period relative risks for incidence and mortality rate (period effects) were calculated as the ratios of age-specific rates in each period group, with 1992–1996 (median, 1994) as the reference period. The period 1992–1996 (median, 1994) and birth cohort 1960–1964 (median, 1962) were selected as reference points based on established methodological principles. These mid-range values chosen to minimize the correlation between period and cohort effects, following recommended practices in age-period-cohort analysis, while providing a central benchmark for relative risk comparisons.

## Results

### Burdens of incidence and mortality, 1990–2021

For all six types of early-onset gastrointestinal cancers, there were 169,117 new cases and 134,196 deaths in 1990, and 208,414 new cases and 107,007 deaths in 2021 (Supplementary Tables S1, S2). In 2021, the number of new cases due to colorectal cancer was the highest (males: 55,926; females: 22,767), followed by gastric (males: 43,483; females: 14,637), liver (males: 33,413; females: 5096), esophageal (males: 16,701; females: 2198), pancreatic (males: 7835; females: 2466), and gallbladder cancer (males: 2615; females: 1279) (Supplementary Table S1 and Supplementary Figure S1). Deaths due to gastric cancer were highest (males: 22,756; females: 7813), followed by liver (males: 24,420; females: 3707), colorectal (males: 17,809; females: 6667), esophageal (males: 11,857; females: 1146), pancreatic (males: 6787; females: 2100), and gallbladder cancer (males: 1270; females: 766) in 2021 (Supplementary Table S2 and Supplementary Figure S1).

The male-to-female ratio increased substantially during the past three decades (1990 *vs*. 2021, esophageal: 5.38 *vs*. 7.60; gastric: 2.20 *vs*. 2.97; colorectal: 1.46 *vs*. 2.46; liver: 4.51 *vs*. 6.56; gallbladder: 1.33 *vs*. 2.04; and pancreatic: 2.39 *vs*. 3.18) (Supplementary Tables S1–S8). The mortality-to-incidence ratio was highest for pancreatic cancer (0.86) in 2021, followed by liver (0.73), esophageal (0.69), gastric (0.53), gallbladder (0.50), and colorectal cancer (0.31) (Supplementary Table S9). Mortality-to-incidence ratio showed an increasing trend in line with increasing age for all six early-onset cancers in 2021, and all were comparable between males and females, except for esophageal cancer (males: 0.71; females: 0.52).

The incidence rate (per 100,000) in 2021 was highest for colorectal cancer (11.86; 95% UI, 9.45 to 14.54), followed by gastric (8.76; 95% UI, 6.92 to 11.22), liver (5.81; 95% UI, 4.60 to 7.57), esophageal (2.85; 95% UI, 2.21 to 3.56), pancreatic (1.55; 95% UI, 1.20 to 1.94), and gallbladder cancer (0.59; 95% UI, 0.36 to 0.77) (Figure 2). The mortality rate (per 100,000) in 2021 was the highest for gastric cancer (4.61; 95% UI, 3.62 to 5.96), followed by liver (4.24; 95% UI, 3.37 to 5.51), colorectal (3.69; 95% UI, 2.94 to 4.52), esophageal (1.96; 95% UI, 1.51 to 2.49), pancreatic (1.34; 95% UI, 1.03 to 1.68), and gallbladder cancer (0.29; 95% UI, 0.19 to 0.38). The incidence and mortality rates were higher in males than females for all six early-onset cancers in 2021. The incidence and mortality rates in 2021 increased largely with increasing age in all six cancers (Supplementary Tables S3-S8 and Supplementary Figures S2, S3).

**Figure 2:**
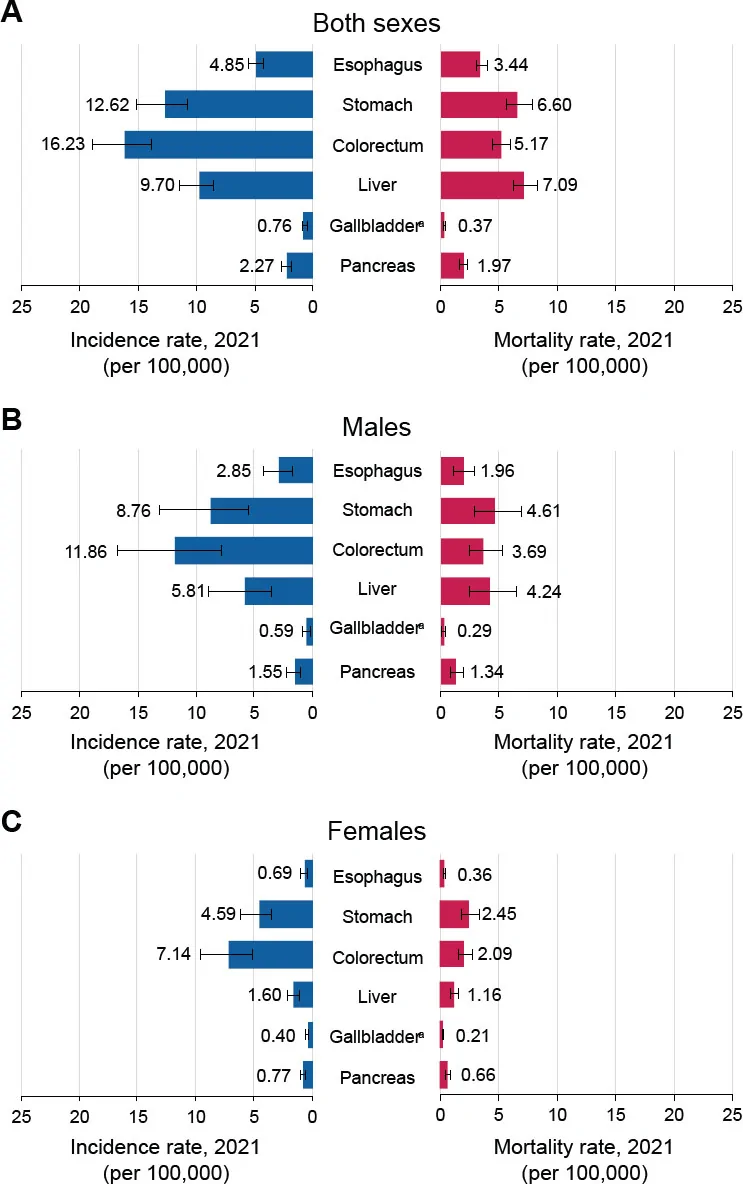
Incidence and mortality rates of early-onset gastrointestinal cancers, 2021. (A) Both sexes, (B) males, (C) females. *Gallbladder and biliary tract cancers.

### Joinpoint regression analysis of temporal trends

We analyzed the temporal trends in incidence and mortality rates from 1990–2021 (Figure 3 and Supplementary Table S10, S11) and, to better understand recent trends, also conducted a focused analysis for 2010–2021 (Supplementary Figure S4, S5 and Supplementary Table S12, S13). Over the entire period (1990–2021), incidence rates increased for colorectal (EAPC, 2.67% per year; 95% CI, 2.43% to 2.91%), gallbladder (EAPC, 2.37% per year; 95% CI, 2.24% to 2.51%), pancreatic cancer (EAPC, 1.64% per year; 95% CI, 1.51% to 1.76%), and liver (EAPC, 0.52% per year; 95% CI, 0.16% to 0.87%), while rates decreased for esophageal (EAPC, –1.51% per year; 95% CI, –1.74% to –1.27%) and gastric cancers (EAPC, –0.75% per year; 95% CI, –0.87% to –0.64%). The analysis of the recent decade (2010–2021) revealed an accelerated increase in incidence trends for colorectal (EAPC, 3.53% per year; 95% CI, 3.34% to 3.71%) and liver (EAPC, 1.08% per year; 95% CI, 0.55% to 1.61%), whereas the increasing trend for pancreatic cancers (EAPC, 1.32% per year; 95% CI, 1.19% to 1.44%) and gallbladder cancer (EAPC, 1.86% per year; 95% CI, 1.65% to 2.07%) moderated. Declines in esophageal (EAPC, –3.52% per year; 95% CI, –4.48% to –2.54%) and gastric cancer incidence (EAPC, –1.01% per year; 95% CI, –1.49% to –0.52%) also intensified during this recent period. The temporal trends in incidence and mortality rates in both sexes and different age groups were largely consistent with the trends in the overall population (Supplementary Tables S10-S13 and Supplementary Figures S4, S5).

**Figure 3:**
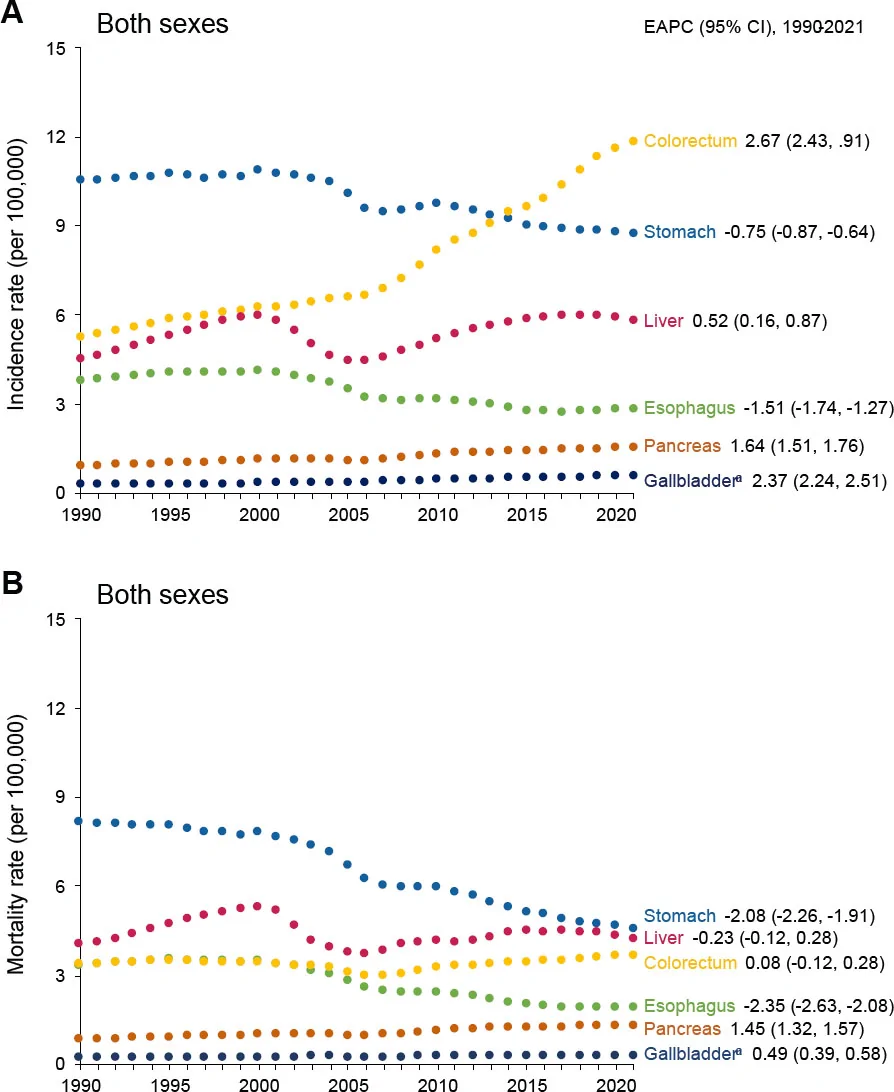
Trends in early-onset gastrointestinal cancers, 1990–2021. (A) Incidence rate, (B) mortality rate. *Gallbladder, gallbladder and biliary tract cancers; EAPC: estimated annual percentage change. EAPC values with 95% confidence intervals (CIs) were analyzed for incidence and mortality rates by Joinpoint regression.

### Age distribution in relation to incidence and deaths, 1990–2021

The proportions of incidence and deaths in each age group were presented for all six cancers during 1990–2021. The proportion of incidence in younger age groups was markedly lower than that in older age groups for all six cancers, although the proportion fluctuated over time (Figure 4). The proportion of deaths was largely consistent with the incidence for all six early-onset cancers (Figure 5). Furthermore, the proportions of incidence and deaths were comparable between males and females for each year during 1990–2021 (Supplementary Figures S6-S9).

**Figure 4:**
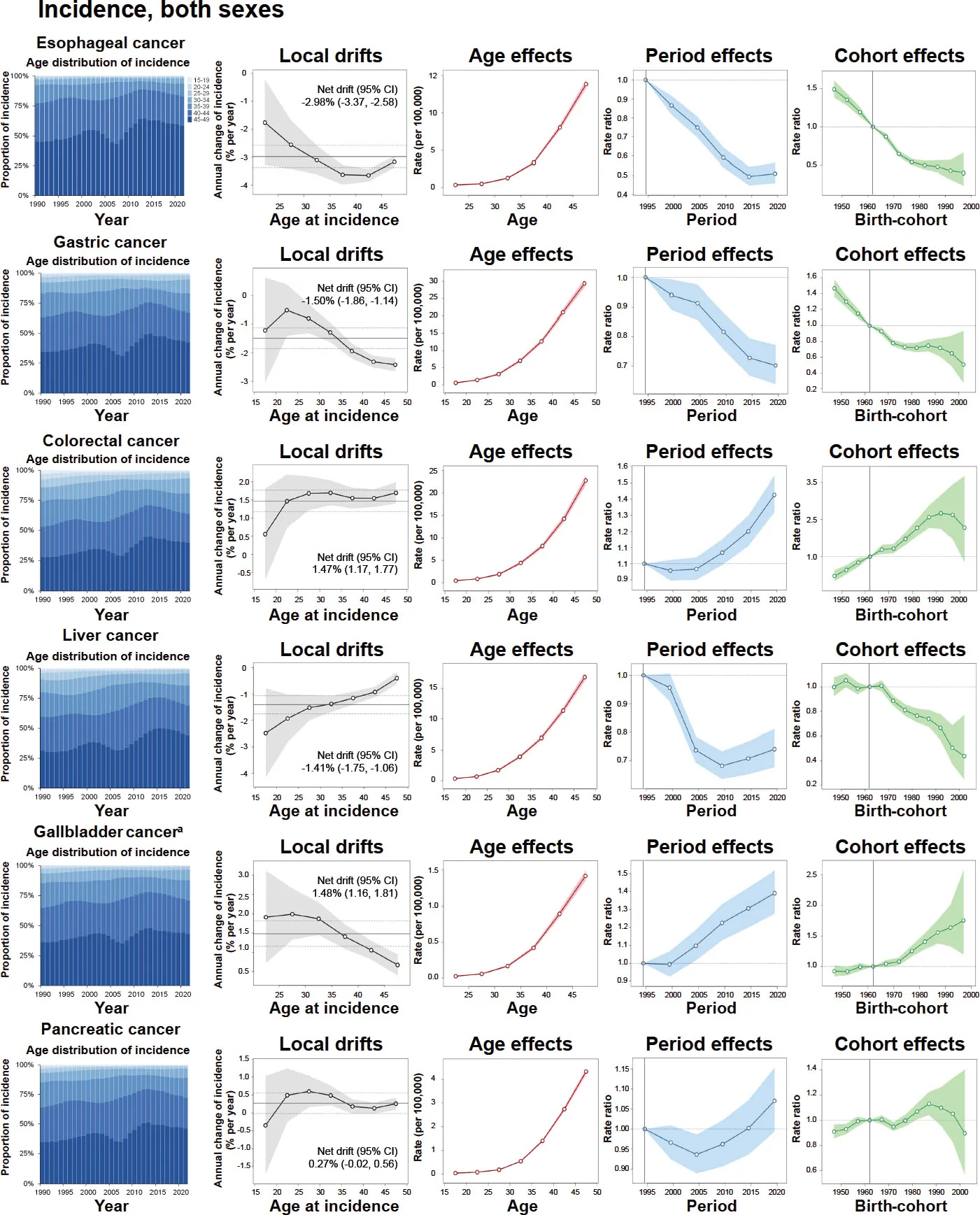
Age-period-cohort analysis of incidence of early-onset gastrointestinal cancers in both sexes. Age distribution of incidence shows relative proportion of incidence in each age group during 1992–2021. Local drift indicates annual percentage change in incidence (% per year) across 5-year age groups from 15–19 (first dot) to 45–49 (last dot) years, and net drift (horizontal line) indicates annual percentage change in incidence (% per year) for the whole population. Age effects represented by fitted longitudinal age curves of incidence adjusted for period deviations, and Y-axis represents expected age-specific rates (per 100,000) adjusted for period effects. Period effects represented by relative risk of incidence (incidence rate ratio) and computed as ratio of age-specific rates in each period compared with the reference period (1992–1996, first dot). Birth cohort effects represented by relative risk of incidence (incidence rate ratio) and computed as ratio of age-specific rates in each cohort compared with the reference birth cohort (1960–1964, fourth dot). Shaded areas indicate corresponding 95% confidence intervals (CIs) of each point estimate. aGallbladder and biliary tract cancers. For esophageal cancer and gallbladder and biliary tract cancer populations, we only included patients aged 20–49 years because no incidence or mortality data were available for 15–19-year-olds in the GBD 2021 study.

**Figure 5:**
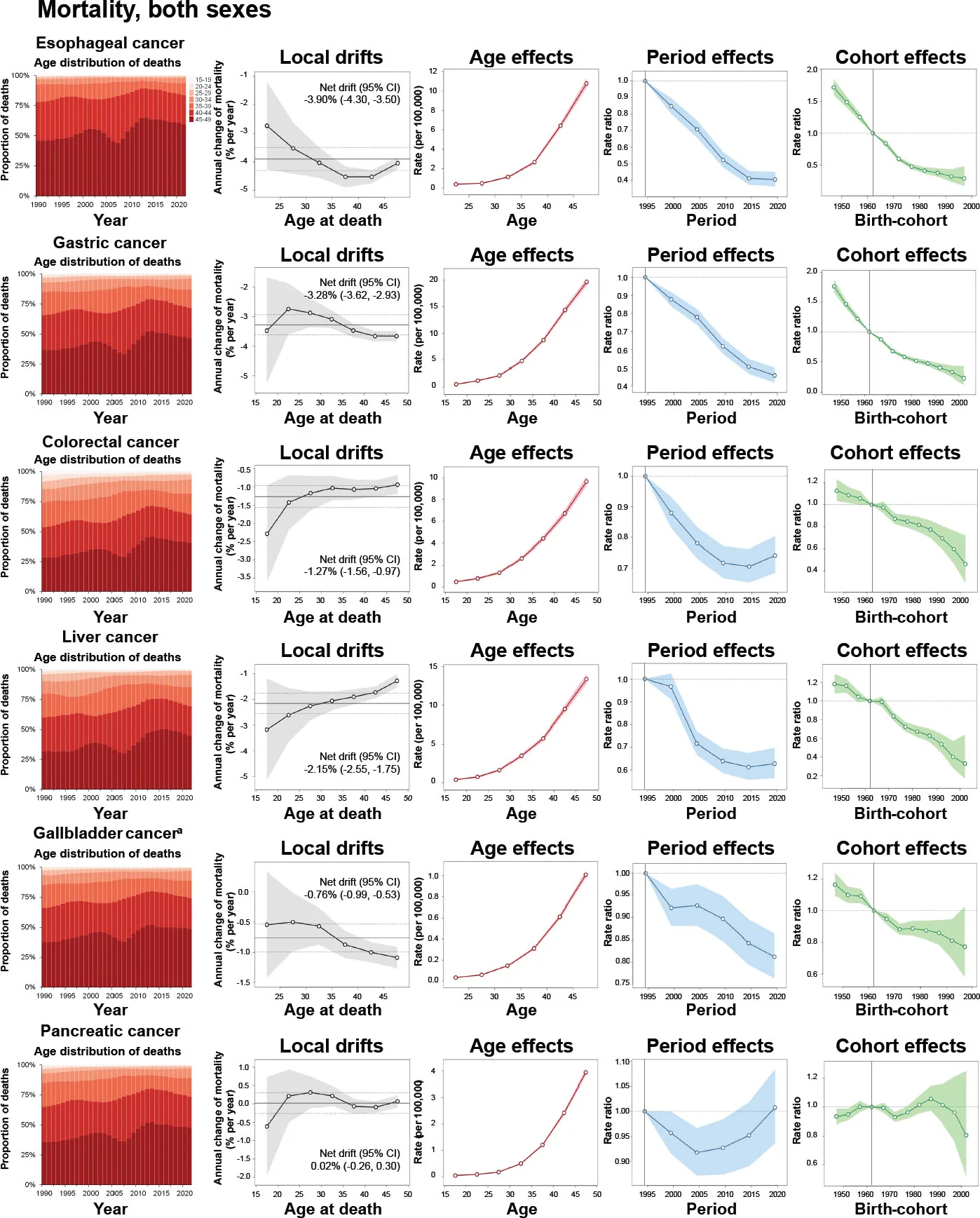
Age-period-cohort analysis of mortality for early-onset gastrointestinal cancers in both sexes. Age distribution of mortality show relative proportion of deaths in each age group during 1992–2021. Local drift indicates annual percentage change in mortality (% per year) across the 5-year age groups 15–19 (first dot) to 45–49 (last dot) years, and net drift (horizontal line) indicates annual percentage change in mortality (% per year) for the whole population. Age effects represented by fitted longitudinal age curves of mortality adjusted for period deviations, and Y-axis represents expected age-specific rates (per 100,000) adjusted for period effects. Period effects represented by relative risk of mortality (mortality rate ratio) and computed as ratio of age-specific rates in each period compared with the reference period (1992–1996, first dot). Birth cohort effects represented by relative risk of mortality (mortality rate ratio) and computed as ratio of age-specific rates in each cohort compared with the reference birth cohort (1956–1964, fourth dot). Shaded areas indicate corresponding 95% confidence intervals (CIs) of each point estimate. *Gallbladder and biliary tract cancers. For esophageal cancer and gallbladder and biliary tract cancer populations, we only included patients aged 20–49 years because no incidence or mortality data were available for 15–19-year-olds in the GBD 2021 study.

### Net and local drifts in different age groups

During 1992–2021, the net drifts reflected the overall annual percentage change in incidence rate, showing an increase in gallbladder cancer (net drift, 1.48%; 95% CI, 1.16% to 1.81%) and colorectal cancer (net drift, 1.47%; 95% CI, 1.17% to 1.77%), a rapid decrease in esophageal (net drift, –2.98%; 95% CI, –3.37% to –2.58%), a slight decreases in gastric cancer (net drift, –1.50%; 95% CI, –1.86% to –1.14%) and liver cancer (net drift, –1.41%; 95% CI, –1.75% to –1.06%), and stable rates in pancreatic (net drift, 0.27%; 95% CI, –0.02% to 0.56%) (Table 1, Figure 4). Local drifts reflected the annual percentage change in incidence rate for each age group relative to the net drift, with variations across different age groups and between sexes (Table 1, Supplementary Tables S14, S15 and Supplementary Figures S6, S7). The incidence rates in males increased for pancreatic cancer in all age groups, but decreased in females.

**Table 1:** Age-period-cohort analysis of incidence of early-onset gastrointestinal cancers

Both sexes	Age-period-cohort analysis for incidence					
	Esophageal cancer	Gastric cancer	Colorectal cancer	Liver cancer	Gallbladder cancer^a^	Pancreatic cancer
Net drifts^b^	-2.98 (-3.37, -2.58)	-1.50 (-1.86, -1.14)	1.47 (1.17, 1.77)	-1.41 (-1.75, -1.06)	1.48 (1.16, 1.81)	0.27 (-0.02, 0.56)
Males	-2.81 (-3.24, -2.37)	-1.16 (-1.61, -0.72)	2.23 (1.86, 2.60)	-1.14 (-1.51, -0.78)	2.39 (2.01, 2.77)	0.78 (0.41, 1.14)
Females	-3.66 (-3.95, -3.38)	-2.12 (-2.41, -1.84)	0.13 (-0.10, 0.37)	-2.55 (-2.84, -2.27)	0.05 (-0.17, 0.26)	-0.90 (-1.10, -0.70)
Local drifts^c^						
15-19 years	NA	-1.23 (-3.03, 0.61)	0.55 (-0.69, 1.81)	-2.48 (-4.14, -0.79)	NA	-0.35 (-1.70, 1.02)
20-24 years	-1.76 (-3.27, -0.23)	-0.52 (-1.41, 0.38)	1.47 (0.74, 2.20)	-1.93 (-2.82, -1.02)	1.91 (0.72, 3.12)	0.49 (-0.25, 1.24)
25-29 years	-2.55 (-3.42, -1.68)	-0.81 (-1.32, -0.30)	1.68 (1.22, 2.15)	-1.52 (-1.99, -1.04)	1.99 (1.33, 2.65)	0.60 (0.17, 1.04)
30-34 years	-3.10 (-3.61, -2.58)	-1.30 (-1.63, -0.96)	1.70 (1.36, 2.03)	-1.38 (-1.68, -1.08)	1.87 (1.45, 2.30)	0.49 (0.21, 0.76)
35-39 years	-3.62 (-3.97, -3.28)	-1.95 (-2.21, -1.69)	1.55 (1.27, 1.83)	-1.16 (-1.40, -0.92)	1.41 (1.09, 1.72)	0.18 (-0.02, 0.38)
40-44 years	-3.65 (-3.87, -3.43)	-2.32 (-2.52, -2.11)	1.55 (1.31, 1.79)	-0.93 (-1.12, -0.74)	1.06 (0.82, 1.30)	0.13 (-0.02, 0.28)
45-49 years	-3.16 (-3.36, -2.96)	-2.42 (-2.65, -2.20)	1.69 (1.40, 1.99)	-0.41 (-0.63, -0.19)	0.67 (0.40, 0.95)	0.26 (0.09, 0.43)
Age effects^d^						
15-19 years	NA	0.60 (0.49, 0.73)	0.49 (0.41, 0.57)	0.36 (0.30, 0.43)	NA	0.04 (0.04, 0.05)
20-24 years	0.36 (0.30, 0.43)	1.43 (1.26, 1.63)	0.89 (0.79, 1.01)	0.70 (0.62, 0.79)	0.03 (0.02, 0.03)	0.08 (0.07, 0.09)
25-29 years	0.52 (0.45, 0.59)	3.15 (2.90, 3.43)	1.92 (1.76, 2.10)	1.73 (1.61, 1.87)	0.06 (0.05, 0.07)	0.18 (0.17, 0.20)
30-34 years	1.28 (1.19, 1.39)	7.06 (6.67, 7.48)	4.43 (4.15, 4.73)	3.92 (3.72, 4.14)	0.17 (0.16, 0.18)	0.54 (0.52, 0.57)
35-39 years	3.32 (3.15, 3.50)	12.69 (12.12, 13.29)	8.19 (7.75, 8.65)	6.97 (6.67, 7.28)	0.42 (0.40, 0.45)	1.40 (1.36, 1.45)
40-44 years	8.03 (7.69, 8.38)	21.01 (20.17, 21.89)	14.25 (13.56, 14.98)	11.37 (10.93, 11.83)	0.90 (0.85, 0.94)	2.74 (2.66, 2.83)
45-49 years	13.84 (13.28, 14.42)	29.35 (28.21, 30.54)	22.74 (21.72, 23.81)	16.82 (16.20, 17.47)	1.43 (1.36, 1.49)	4.32 (4.20, 4.45)
Period effects^e^						
1992-1996	1.00	1.00	1.00	1.00	1.00	1.00
1997-2001	0.87 (0.82, 0.92)	0.94 (0.89, 0.99)	0.96 (0.90, 1.02)	0.96 (0.91, 1.01)	0.99 (0.93, 1.07)	0.97 (0.92, 1.01)
2002-2006	0.75 (0.70, 0.81)	0.91 (0.86, 0.97)	0.97 (0.90, 1.04)	0.74 (0.69, 0.78)	1.10 (1.02, 1.19)	0.94 (0.89, 0.99)
2007-2011	0.59 (0.54, 0.64)	0.82 (0.76, 0.88)	1.07 (0.99, 1.15)	0.68 (0.64, 0.73)	1.22 (1.13, 1.33)	0.96 (0.91, 1.02)
2012-2016	0.49 (0.45, 0.55)	0.73 (0.67, 0.79)	1.20 (1.11, 1.30)	0.71 (0.65, 0.77)	1.31 (1.20, 1.42)	1.00 (0.94, 1.07)
2017-2021	0.51 (0.46, 0.57)	0.70 (0.64, 0.77)	1.43 (1.32, 1.54)	0.74 (0.68, 0.81)	1.39 (1.28, 1.51)	1.07 (0.99, 1.15)
Birth cohort effect^e^						
1945-1949	1.49 (1.38, 1.60)	1.47 (1.36, 1.58)	0.74 (0.67, 0.82)	1.00 (0.93, 1.08)	0.92 (0.84, 1.02)	0.91 (0.86, 0.96)
1950-1954	1.35 (1.28, 1.43)	1.30 (1.23, 1.37)	0.82 (0.77, 0.88)	1.05 (1.00, 1.11)	0.92 (0.86, 0.99)	0.93 (0.89, 0.97)
1955-1959	1.19 (1.13, 1.25)	1.15 (1.09, 1.20)	0.92 (0.87, 0.98)	0.99 (0.94, 1.04)	0.99 (0.93, 1.06)	0.99 (0.95, 1.03)
1960-1964	1.00	1.00	1.00	1.00	1.00	1.00
1965-1969	0.87 (0.83, 0.92)	0.93 (0.89, 0.97)	1.10 (1.04, 1.16)	1.01 (0.96, 1.05)	1.04 (0.99, 1.10)	1.00 (0.97, 1.04)
1970-1974	0.65 (0.62, 0.69)	0.78 (0.74, 0.82)	1.11 (1.05, 1.17)	0.89 (0.85, 0.93)	1.08 (1.02, 1.14)	0.95 (0.92, 0.98)
1975-1979	0.54 (0.50, 0.59)	0.73 (0.69, 0.78)	1.24 (1.16, 1.32)	0.81 (0.77, 0.86)	1.25 (1.17, 1.34)	1.00 (0.95, 1.04)
1980-1984	0.50 (0.45, 0.56)	0.72 (0.67, 0.78)	1.39 (1.28, 1.50)	0.76 (0.71, 0.82)	1.41 (1.28, 1.54)	1.07 (1.01, 1.13)
1985-1989	0.48 (0.41, 0.57)	0.75 (0.68, 0.83)	1.53 (1.39, 1.68)	0.74 (0.67, 0.81)	1.55 (1.38, 1.76)	1.13 (1.04, 1.22)
1990-1994	0.43 (0.32, 0.57)	0.72 (0.61, 0.84)	1.58 (1.37, 1.83)	0.67 (0.57, 0.78)	1.63 (1.33, 2.01)	1.10 (0.96, 1.26)
1995-1999	0.41 (0.25, 0.67)	0.65 (0.49, 0.87)	1.56 (1.24, 1.97)	0.51 (0.38, 0.69)	1.75 (1.20, 2.56)	1.05 (0.83, 1.33)
2000-2004	NA	0.51 (0.28, 0.93)	1.39 (0.93, 2.08)	0.44 (0.25, 0.77)	NA	0.90 (0.57, 1.40)

NA, not available.

a Gallbladder and biliary tract cancers.

b Net drift indicates annual percentage change in incidence (% per year) for the whole population, with corresponding 95% confidence intervals.

c Local drift indicates annual percentage change in incidence (% per year) for each age group, with their corresponding 95% confidence intervals.

d Expected age-specific rates (per 100,000) and 95% confidence intervals adjusted for period effects.

e Relative risk with 95% confidence intervals.

The overall annual percentage changes in mortality rates during 1990–2021 showed substantial decreases in esophageal (net drift, -3.90%; 95% CI, -4.30% to -3.50%), gastric (net drift, -3.28%; 95% CI, -3.62% to -2.93%) and liver cancer (net drift, -2.15%; 95% CI, -2.55% to -1.75%), a slight decrease in colorectal (net drift, -1.27%; 95% CI, -1.56% to -0.97%) and gallbladder cancer (net drift, -0.76%; 95% CI, -0.99% to -0.53%), and stable in pancreatic cancer (net drift, 0.02%; 95% CI, -0.26% to 0.30%) (Table 2, Figure 5). Local drifts exhibited large differences in mortality rates across different age groups and between sexes (Table 2, Supplementary Tables S16, S17 and Supplementary Figures S8, S9). The mortality rate for pancreatic cancer in patients aged 25–49 years showed an overall increasing trend in males but a decrease in females.

**Table 2:** Age-period-cohort analysis of mortality for early-onset gastrointestinal cancers

Both sexes	Age-period-cohort analysis for mortality					
	Esophageal cancer	Gastric cancer	Colorectal cancer	Liver cancer	Gallbladder cancer^a^	Pancreatic cancer
Net drifts^b^	-3.90 (-4.30, -3.50)	-3.28 (-3.62, -2.93)	-1.27 (-1.56, -0.97)	-2.15 (-2.55, -1.75)	-0.76 (-0.99, -0.53)	0.02 (-0.26, 0.30)
Males	-3.64 (-4.08, -3.21)	-2.95 (-3.40, -2.50)	-0.49 (-0.85, -0.12)	-1.88 (-2.30, -1.46)	0.06 (-0.23, 0.35)	0.54 (0.18, 0.89)
Females	-5.25 (-5.54, -4.96)	-3.85 (-4.09, -3.61)	-2.73 (-2.96, -2.51)	-3.33 (-3.66, -3.00)	-1.98 (-2.25, -1.72)	-1.20 (-1.41, -0.99)
Local drifts^c^						
15-19 years	NA	-3.48 (-5.25, -1.67)	-2.29 (-3.60, -0.96)	-3.18 (-5.12, -1.19)	NA	-0.62 (-1.94, 0.73)
20-24 years	-2.74 (-4.26, -1.20)	-2.74 (-3.60, -1.88)	-1.43 (-2.17, -0.68)	-2.61 (-3.65, -1.56)	-0.55 (-1.41, 0.33)	0.21 (-0.51, 0.94)
25-29 years	-3.53 (-4.41, -2.63)	-2.87 (-3.35, -2.39)	-1.17 (-1.64, -0.71)	-2.26 (-2.81, -1.70)	-0.50 (-0.98, -0.02)	0.31 (-0.12, 0.74)
30-34 years	-4.03 (-4.56, -3.50)	-3.09 (-3.39, -2.79)	-1.03 (-1.36, -0.71)	-2.06 (-2.42, -1.70)	-0.57 (-0.87, -0.27)	0.21 (-0.06, 0.49)
35-39 years	-4.53 (-4.88, -4.16)	-3.47 (-3.70, -3.24)	-1.07 (-1.34, -0.80)	-1.90 (-2.18, -1.61)	-0.87 (-1.09, -0.65)	-0.07 (-0.27, 0.13)
40-44 years	-4.52 (-4.75, -4.30)	-3.66 (-3.83, -3.49)	-1.04 (-1.27, -0.82)	-1.73 (-1.95, -1.50)	-1.00 (-1.16, -0.83)	-0.09 (-0.24, 0.06)
45-49 years	-4.05 (-4.25, -3.84)	-3.66 (-3.84, -3.48)	-0.94 (-1.20, -0.68)	-1.28 (-1.53, -1.02)	-1.09 (-1.26, -0.91)	0.07 (-0.10, 0.23)
Age effects^d^						
15-19 years	NA	0.56 (0.47, 0.66)	0.49 (0.43, 0.57)	0.39 (0.32, 0.48)	NA	0.04 (0.04, 0.05)
20-24 years	0.38 (0.32, 0.45)	1.23 (1.11, 1.37)	0.80 (0.71, 0.89)	0.76 (0.66, 0.86)	0.03 (0.03, 0.04)	0.09 (0.08, 0.10)
25-29 years	0.48 (0.42, 0.55)	2.20 (2.04, 2.36)	1.33 (1.22, 1.45)	1.63 (1.49, 1.79)	0.06 (0.06, 0.06)	0.17 (0.16, 0.19)
30-34 years	1.13 (1.05, 1.23)	4.80 (4.57, 5.04)	2.63 (2.48, 2.80)	3.51 (3.30, 3.73)	0.15 (0.14, 0.15)	0.50 (0.47, 0.52)
35-39 years	2.66 (2.53, 2.81)	8.71 (8.38, 9.04)	4.41 (4.19, 4.64)	5.73 (5.45, 6.04)	0.31 (0.30, 0.32)	1.20 (1.16, 1.25)
40-44 years	6.43 (6.16, 6.71)	14.35 (13.88, 14.84)	6.70 (6.40, 7.03)	9.48 (9.05, 9.93)	0.61 (0.59, 0.63)	2.42 (2.35, 2.50)
45-49 years	10.77 (10.33, 11.23)	19.64 (19.01, 20.30)	9.64 (9.22, 10.09)	13.35 (12.77, 13.96)	1.01 (0.97, 1.04)	3.93 (3.82, 4.05)
Period effects^e^						
1992-1996	1.00	1.00	1.00	1.00	1.00	1.00
1997-2001	0.85 (0.80, 0.90)	0.88 (0.84, 0.92)	0.88 (0.83, 0.93)	0.97 (0.91, 1.02)	0.92 (0.88, 0.96)	0.96 (0.92, 1.00)
2002-2006	0.71 (0.66, 0.76)	0.78 (0.74, 0.82)	0.78 (0.73, 0.83)	0.72 (0.67, 0.77)	0.93 (0.88, 0.98)	0.92 (0.87, 0.97)
2007-2011	0.52 (0.48, 0.57)	0.62 (0.58, 0.66)	0.72 (0.67, 0.77)	0.64 (0.59, 0.69)	0.90 (0.85, 0.95)	0.93 (0.87, 0.98)
2012-2016	0.41 (0.37, 0.46)	0.50 (0.47, 0.55)	0.71 (0.65, 0.76)	0.61 (0.56, 0.67)	0.84 (0.79, 0.90)	0.95 (0.89, 1.02)
2017-2021	0.41 (0.37, 0.45)	0.46 (0.42, 0.50)	0.74 (0.69, 0.80)	0.63 (0.57, 0.70)	0.81 (0.76, 0.86)	1.01 (0.94, 1.08)
Birth cohort effect^f^						
1945-1949	1.72 (1.60, 1.84)	1.76 (1.66, 1.87)	1.13 (1.03, 1.23)	1.18 (1.08, 1.29)	1.16 (1.10, 1.24)	0.93 (0.88, 0.99)
1950-1954	1.48 (1.40, 1.57)	1.47 (1.40, 1.53)	1.09 (1.02, 1.16)	1.16 (1.09, 1.24)	1.10 (1.05, 1.15)	0.95 (0.91, 0.99)
1955-1959	1.25 (1.19, 1.32)	1.22 (1.17, 1.27)	1.06 (1.00, 1.12)	1.04 (0.99, 1.11)	1.09 (1.05, 1.14)	1.00 (0.96, 1.04)
1960-1964	1.00	1.00	1.00	1.00	1.00	1.00
1965-1969	0.84 (0.80, 0.88)	0.87 (0.84, 0.91)	0.97 (0.92, 1.03)	0.99 (0.94, 1.05)	0.95 (0.91, 0.98)	0.99 (0.96, 1.03)
1970-1974	0.60 (0.56, 0.63)	0.68 (0.65, 0.71)	0.87 (0.83, 0.92)	0.83 (0.79, 0.88)	0.88 (0.85, 0.92)	0.93 (0.90, 0.96)
1975-1979	0.47 (0.44, 0.51)	0.58 (0.55, 0.61)	0.85 (0.79, 0.91)	0.72 (0.68, 0.77)	0.89 (0.84, 0.93)	0.96 (0.92, 1.01)
1980-1984	0.41 (0.37, 0.47)	0.52 (0.48, 0.55)	0.82 (0.75, 0.89)	0.67 (0.62, 0.73)	0.87 (0.82, 0.94)	1.02 (0.96, 1.08)
1985-1989	0.38 (0.32, 0.45)	0.47 (0.43, 0.52)	0.78 (0.70, 0.86)	0.63 (0.56, 0.70)	0.86 (0.78, 0.94)	1.06 (0.97, 1.15)
1990-1994	0.32 (0.24, 0.43)	0.40 (0.34, 0.47)	0.70 (0.60, 0.81)	0.54 (0.45, 0.65)	0.81 (0.70, 0.94)	1.01 (0.88, 1.16)
1995-1999	0.29 (0.17, 0.48)	0.33 (0.24, 0.43)	0.60 (0.47, 0.77)	0.40 (0.28, 0.57)	0.77 (0.58, 1.03)	0.96 (0.76, 1.21)
2000-2004	NA	0.23 (0.12, 0.42)	0.46 (0.30, 0.72)	0.33 (0.17, 0.64)	NA	0.81 (0.52, 1.25)

NA, not available.

a Gallbladder and biliary tract cancers.

b Net drift indicates annual percentage change in mortality (% per year) for the whole population, with their corresponding 95% confidence intervals.

c Local drift indicates annual percentage change in mortality (% per year) for each age group, with their corresponding 95% confidence intervals.

d Expected age-specific rates (per 100,000) and 95% confidence intervals adjusted for period effects.

e Relative risk with 95% confidence intervals.

### Age effects on incidence and mortality

There were sustained increases in the incidence and mortality risks for all six early-onset cancers in line with increasing age, with substantial variations (Tables 1, 2 and Figures 4, 5). Compared with the expected age-specific incidence rate (adjusted for period effects) in the 20–24 age group, the incidence rates in the 45–49 age group increased 54-fold (pancreatic cancer), 48-fold (gallbladder cancer), 38-fold (esophageal cancer), 26-fold (colorectal cancer), 24-fold (liver cancer), and 21-fold (gastric cancer). The expected age-specific mortality rates in the 45–49 age group increased 44-fold (pancreatic cancer), 34-fold (gallbladder cancer), 28-fold (esophageal cancer), 18-fold (liver cancer), 16-fold (gastric cancer), and 12-fold (colorectal cancer), compared with the 20–24 age group. The age effects on incidence and mortality were greater in males than females for all six early-onset cancers (Supplementary Tables S14–S17 and Supplementary Figures S6–S9).

### Period effects on incidence and mortality risk

The period effects on incidence risk varied among the six cancers (Table 1 and Figure 4). For esophageal, gastric and liver cancers, the period effects on incidence risk decreased continuously from 1997–2001 to 2017–2021. Specifically, the relative risks for the most recent period (2017–2021) were significantly less than 1, as exemplified by esophageal cancer (relative risk: 0.51, 95% CI: 0.46 to 0.57), indicating a substantially lower risk compared to the reference period (1992–1996). In contrast, for colorectal and gallbladder cancers, the period effects decreased during 1997–2001 but increased from 2007–2011 to 2017–2021. The period effects remained roughly constant for pancreatic cancer over the entire period. The period effect on incidence risk showed large variations between males and females for all six cancers (Supplementary Tables S14, S15 and Supplementary Figures S6, S7).

Regarding the mortality, the period effect for gastric cancer demonstrated a decreasing impact throughout the entire period (Table 2 and Figure 5). For esophageal and liver cancers, the period effect on mortality risk decreased rapidly from 1997–2001 to 2007–2011, while for colorectal cancer, it remained stable from 2007–2011 to 2017–2021. The period effect for gallbladder cancer showed improvement over the last two decades (2002–2006 to 2017–2021), in contrast to the upward trend observed for pancreatic cancer. There were wide variations in the period effects on mortality risk between males and females for all six cancers (Supplementary Tables S16, S17 and Supplementary Figures S8, S9).

### Birth cohort effects on incidence and mortality risk

The birth cohort effects on the incidence risk varied in all six early-onset cancers (Table 1 and Figure 4). Overall declining trends in incidence risk were observed in successively younger birth cohorts for esophageal and gastric cancers, while rising birth-cohort trends were seen for colorectal and gallbladder cancers. For colorectal cancer, successive cohorts born after the 1960s exhibited a clear trend of increasing risk, culminating in a relative risk of 1.56 (95% CI: 1.24 to 1.97) for the 1995–1999 birth cohort, significantly greater than 1 and indicative of a strong cohort effect in younger generations. Compared with the 1960–1964 reference cohort, the relative risk of liver cancer for individuals born after 1964 declined from 1.01 (95% CI: 0.96 to 1.05) in the 1965–1969 birth cohort to 0.44 (95% CI: 0.25 to 0.77) in the 2000–2004 birth cohort. The birth-cohort effects on incidence risk remained roughly constant across all cohorts for pancreatic cancer. The birth cohort effects on incidence risks in males and females are shown in Supplementary Tables S14 and S15 and Supplementary Figures S6 and S7.

For mortality risk, overall declining trends in successively younger birth cohorts were observed for colorectal, liver and gallbladder cancers, with this trend being particularly pronounced for esophageal and gastric cancers (Table 2 and Figure 5). Compared with the 1960–1964 reference cohort, the relative risk of pancreatic cancer declined progressively from 1.06 (95% CI: 0.97 to 1.15) in the 1985–1989 cohort to 0.81 (95% CI: 0.52 to 1.25) in the 2000–2004 cohort, suggesting a weakening birth cohort effect on mortality risk in younger generations. Birth cohort effects on the risks of mortality in males and females for all six early-onset cancers are presented in Supplementary Tables S16 and S17 and Supplementary Figures S8 and S9.

### Impact of SDI on Incidence and mortality Rates, 1990–2021

The relationship between the SDI and the incidence and mortality rates of six major types of early-onset gastrointestinal cancers in China is illustrated in Supplementary Figure S10. Overall, distinct patterns were observed across different cancer types. The incidence of esophageal and gastric cancers exhibited a nonlinear association with SDI, with an initial increase observed at lower SDI levels and a subsequent decline after reaching a peak at a moderate level of approximately 0.55. In contrast, the incidence rates of colorectal, gallbladder, and pancreatic cancers showed a continuous upward trend with increasing SDI. Regarding mortality, esophageal and gastric cancer rates decreased with rising SDI, while pancreatic cancer mortality increased significantly. Both incidence and mortality rates of liver cancer demonstrated fluctuating patterns in relation to SDI.

## Discussion

From 1990 to 2021, there were a total of 6,520,912 new cases and 4,321,380 deaths due to early-onset gastrointestinal cancers in China. As expected, the incidence and number of deaths due to early-onset gastrointestinal cancers were higher in males than in females, with a substantial increase in the male-to-female ratio during the past three decades. The sex disparities reported here are primarily based on descriptive comparisons of age-standardized rates and male-to-female ratios. While these marked and persistent differences strongly suggest important underlying biological and behavioral risk factors, formal causal attribution requires future individual-level studies. There were substantial differences in recent temporal trends across the six major types of early-onset gastrointestinal cancers. The mortality rates of colorectal, liver, gallbladder, and pancreatic cancers rose gradually from 2010 to 2021, whereas those of esophageal and gastric cancers decreased gradually. To the best of our knowledge, this study provided the first estimate of the latest temporal trends in incidence and mortality, as well as assessments of the age, period, and birth cohort effects, for six major types of early-onset gastrointestinal cancers in China. Local drifts are crucial to understanding the temporal trends for each age group,^[24]^ while the period and birth cohort effects enabled us to distinguish the sources of the incidence and mortality trends in terms of temporal period and birth cohort, thus providing helpful information for gastrointestinal cancer-related health care services in China. The findings of this study carry significant implications for clinical practice and public health policy in China. By disentangling the age, period, and cohort effects, our analysis not only describes past trends but also provides a evidence base for future action. The distinct trajectories observed for different cancer types underscore the need for differentiated and targeted strategies for cancer control among adolescents and young adults.

Its huge population and high prevalence of multiple etiological factors mean that China currently accounts for approximately half of all global esophageal cancer cases and deaths.^[13]^ The incidence of early-onset esophageal cancer in China was increasing until 1996, but then decreased continuously during 1997–2021. Similarly, the period effects on incidence risk decreased sharply from 2005–2001 to 2017–2021, although the reasons for this fall remain largely unknown. Tobacco is a major risk factor for esophageal squamous cell cancer and a moderate risk factor for adenocarcinoma,^[26, 27]^ and Chinese smoking rates notably fell by 22.4% for males and 48.4% for females from 1990 to 2015.^[28]^ The levels of cancer-prevention awareness in China has improved following national public health initiatives targeting cancer prevention and control, *i.e*., the Outline of China Cancer Prevention and Control Plan 2004–2010.

The period effects on the incidence risk of early-onset gastric cancer decreased steadily from the period 1997–2001 to 2017–2021. *Helicobacter pylori* (*H. pylori*) infection is a well-established risk factor for gastric cancer in both Eastern and Western countries, including in younger populations.^[29, 30]^ The decreased period effect on the incidence risk of gastric cancer during the latest decade may thus be largely attributed to the emphasis on endoscopic screening followed by the eradication of *H. pylori*.^[31]^ Although endoscopic screening will not affect the incidence of gastric cancer, it may reduce the associated mortality risk by increasing its early detection.^[32, 33]^ Nationwide endoscopic screening followed by *H. pylori* eradication programs in Japan and Korea have effectively increased the detection rate of early gastric cancer and thus improved the survival rate of gastric cancer patients.^[34, 35]^ China launched a screening program for gastric cancer in high-risk areas in 2006,^[36]^ which may partly explain the decrease in period effects on mortality risk from the period 2007–2011 to 2017–2021 in China. This program improved public awareness of cancer screening in China, and may thus also have contributed to improved survival.^[37]^

The declining trends in gastric and esophageal cancers demonstrate the success of past public health efforts, such as smoking control and endoscopic screening in high-risk areas. This success story argues for both the nationwide expansion and strengthening of these existing interventions and the scaling up of cost-effective *H. pylori* screening and eradication programs beyond high-risk areas, as part of the ‘Healthy China 2030’ agenda. For clinicians, these positive trends validate the importance of early detection through endoscopy. However, the persistent burden, especially in males, highlights the need to improve the accessibility and uptake of screening services and to address remaining risk factors like high salt intake. Continued efforts in patient education and healthcare professional training are essential to consolidate these gains.

There was a continuous and remarkable increase in the incidence rate of early-onset colorectal cancer during 1990–2021, with little evidence for any favorable period or birth cohort effects on cancer risk. Here, we observed similar increasing period and birth cohort effects on the incidence risk of early-onset colorectal cancer from 1997–2001 to 2017–2021. Notably, younger Chinese cohorts are more likely to adopt unhealthy eating behaviors (*i.e*., snacking, eating outside) and to have sedentary behaviors compared with older cohorts,^[38]^ resulting in an increasing incidence of obesity (especially in adolescents), which is a well-known risk factor for early-onset colorectal cancer.^[39, 40, 41]^ In this study, we found stronger period and birth cohort effects on early-onset colorectal cancer incidence and mortality risk among males than females, possibly reflecting the fact that males in China are currently experiencing a higher increase in overweight and obesity than females, as demonstrated in previous studies.^[38, 42, 43]^ These findings have direct implications for clinical practice. The steady rise in incidence and the strong birth cohort effect suggest that the current screening strategy, which typically initiates at age 50 or 55, may be missing a growing at-risk population. Our data support the consideration of lowering the starting age for routine colorectal cancer screening in China, particularly for high-risk individuals (*e.g*., those with a family history, obesity, or unhealthy lifestyle patterns). Clinicians should maintain a higher index of suspicion for colorectal cancer in younger patients presenting with relevant symptoms. From a public health perspective, the rising cohort effect underscores the urgent need for targeted primary prevention strategies aimed at adolescents and young adults. Public health campaigns should be intensified to promote healthy diets rich in fiber, reduce the consumption of processed meats and sugary beverages, and encourage regular physical activity. Integrating nutrition education and physical activity programs into school curricula and workplace wellness initiatives could be effective in curbing the future burden of this largely preventable cancer.

The period effects on the incidence risk of early-onset liver cancer declined during the period 1997–2006, probably attributed to the nationwide hepatitis B vaccination program launched by the Chinese government in 1992. Hepatitis B vaccination has been shown to be crucial in decreasing the risk of liver cancer.^[44, 45]^ The notable decline in the period effect for liver cancer incidence around the early 2000s serves as a powerful testament to the long-term effectiveness of population-level vaccination programs. It provides robust epidemiological evidence supporting the continued investment in and enforcement of universal hepatitis B vaccination as a cornerstone of cancer prevention in China. Implementation of the volunteer blood donation system,^[46]^ decreased exposure to aflatoxin and aristolochic acid,^[47, 48]^ and improved cancer-prevention awareness^[37]^ may also have contributed to the falling incidence rate of liver cancer. The incidence rate of early-onset liver cancer increased continuously during 2007–2021, possibly reflecting changing lifestyles, *i.e*., harmful alcohol consumption, with 57.3% of males and 26.6% of females in China reporting binge drinking.^[49]^ Alcoholic production and consumption have increased steadily in China since 2004, and the average annual alcohol consumption among people aged > 15 years has increased progressively from 2.5 L in 1978 to 4.9 L in 2009.^[50]^ Alcoholic liver cirrhosis is a well-known risk factor for liver cancer, especially among the younger population aged 25–34 years.^[51, 52]^ Furthermore, changing lifestyles (*i.e*., sedentary lifestyle and high-calorie diet) and metabolic disorders (*i.e*., obesity, diabetes, impaired glucose metabolism, metabolic syndrome, non-alcoholic fatty liver disease) in adolescents and adults were also associated with the increased risk of liver cancer.^[53, 54, 55, 56]^

The period effects on the incidence risk of early-onset gallbladder cancer increased continuously from 2002–2006 to 2017–2021 among males, but declined continuously among females. The causes of early-onset gallbladder cancer are not well understood. Gallstones and *Salmonella typhi* carrier state are known major risk factors for adult gallbladder cancer;^[57, 58]^ however, these could not fully explain the substantial difference in period effects between males and females. Notably, obesity is another major contributor to the risk of gallbladder cancer,^[57, 59]^ and the greater increases in obesity and overweight in young males compared with females in China during the period 1985–2010 may partially explain the sex-difference in incidence risks of early-onset gallbladder cancer.^[38, 42]^ Furthermore, high parity and early age at first pregnancy are also risk factors for gallbladder cancer in females.^[60, 61]^ After the launch of China's 1970 policy promoting delayed marriage, extended birth spacing, and reduced fertility and the 1979 policy restricting each couple to a single child, the mean number of children born per woman dropped sharply from 5.9 in 1970 to 1.7 in 2004,^[62]^ and the age at first pregnancy for Chinese females increased by > 6 years from 1950 to 1995,^[63]^ partly explaining the significant reduction in period effects for early-onset gallbladder cancer in females.

The incidence rate of early-onset pancreatic cancer in China increased continuously during 1990–2021, and the period effects on incidence risk increased remarkably over the past decade. Early-onset pancreatic cancer is associated with well-established risk factors including excessive alcohol consumption and obesity.^[64, 65]^ Alcohol consumption (*i.e*., ≥ 26 g/day) could increase the risk of early-onset pancreatic cancer by 49%,^[65]^ and pancreatic cancer at a younger age (< 45 years) was more closely linked to excessive alcohol consumption than at an older age ( ≥ 45 years). In addition, China experienced a significant and continuous increase in the prevalence of obesity in children and adolescents during 1985–2010.^[42]^ These rises in alcohol consumption and obesity, especially during early life, may therefore have played decisive roles in the increases in incidence rates and period effects among early-onset pancreatic cancer patients in China.^[42,43,49,50,66]^ The alarming rise in the birth cohort effect for pancreatic cancer points to the growing impact of early-life risk factors. This underscores that prevention must begin early. Public health strategies need to focus on pediatric and adolescent health to combat childhood obesity and discourage early initiation of alcohol use, thereby altering the disease trajectory for future generations.

There are substantial differences in the epidemiological features of early-onset and later-onset cancers. Epidemiological studies of later-onset gastrointestinal cancers ( ≥ 50 years) therefore fail to reflect the temporal trends in early-onset cancers (< 50 years), and cannot identify the possible causes of the recent rising (*i.e*., early-onset colorectal and liver cancers) or falling trends (*i.e*., early-onset gastric and esophageal cancers).^[67, 68, 69]^ To the best of our knowledge, this study carried out the first systematic analysis of the temporal trends of six major types of early-onset gastrointestinal cancers in China using an age-period-cohort model, to reveal the shifts in incidence and mortality risks for each type of cancer, and to capture significant trends in particular populations by analyzing the effects of age, period, and birth cohort.

Our findings provide critical, up-to-date evidence that can directly inform and refine the cancer control goals of the Healthy China 2030 initiative. This is exemplified in two key aspects: the validation of past successes and the identification of future priorities. Notably, the current results demonstrated the impact of the launch of a specific public health policy, for instance, we noted a remarkable decrease in period effects on the incidence risk of early-onset liver cancer since 2000–2004, after the launch of China's nationwide hepatitis B vaccination program in 1992. Furthermore, the results provided evidence for future policies; *e.g*., an incidence rate > 10 per 100,000 cannot be ignored, and routine gastrointestinal endoscopic screening may be warranted in individuals before the age of 45 years for esophageal cancer, and before 40 years for stomach and colorectal cancer, especially in populations with early-life risk factor exposures (*i.e*., *H. pylori* infection, obesity, alcohol consumption, and smoking), or with a family history of gastrointestinal cancers. To address this rising burden, the next phase of Healthy China 2030 should incorporate targeted actions for younger populations, including launching lifestyle intervention campaigns and evaluating earlier screening strategies.

Our analysis further elucidates the profound influence of China's socioeconomic development, as measured by the SDI, on the evolving burden of early-onset gastrointestinal cancers. The heterogeneous associations between SDI and the incidence and mortality of different cancers underscore a complex epidemiological transition. The initial rise and subsequent decline in the burden of esophageal and gastric cancers after SDI reached approximately 0.55 align with the implementation of nationwide public health interventions, such as endoscopic screening and *H. pylori* eradication programs,^[31, 32, 33]^ which were facilitated by improved economic resources and infrastructure. This trajectory suggests that socioeconomic advancement can mitigate the burden of certain cancers through improved living conditions, enhanced healthcare access, and effective public health policies. Conversely, the steady increase in the burden of colorectal, pancreatic, and gallbladder cancers with rising SDI highlights the unintended consequences of economic development, including the adoption of Westernized lifestyles characterized by dietary changes, reduced physical activity, and rising obesity rates.^[38, 42]^ The fluctuating pattern observed for liver cancer likely reflects the interplay of multiple factors: the beneficial impact of nationwide vaccination programs (*e.g*., hepatitis B) and improved food safety, which initially reduced the burden, being partially offset in recent years by the rising tide of lifestyle risk factors such as alcohol consumption and metabolic syndromes.^[44,45,49, 50, 51, 52]^ These findings collectively emphasize that socioeconomic development is a double-edged sword, simultaneously introducing new risk factors while providing the tools to combat traditional ones. Consequently, future cancer control strategies must be dual-focused: continuing to reinforce successful, targeted public health interventions against traditional risk factors while aggressively implementing policies and education campaigns to address the emerging threats of lifestyle-related risks, particularly among the younger population navigating a rapidly modernizing society.

This study has several limitations. First, the analysis relied solely on the GBD database. While the GBD provides a standardized and comprehensive framework for global comparative studies, its estimates are model-based and may not fully capture subnational variations within China. The lack of direct cross-validation with local cancer registry data are a constraint, primarily due to the scarcity and incompleteness of publicly available data for detailed age-period-cohort analysis across China. Second, the study is ecological in nature, which precludes the analysis of individual-level risk factors, such as genetic predisposition, detailed dietary habits, or specific environmental exposures, which might help explain the observed sex disparities and cohort effects. Third, while our study employed a robust age-period-cohort framework using the National Cancer Institute web tool, we acknowledge that the assessment of model fit relied on visual inspection rather than formal goodness-of-fit statistics. This is an inherent characteristic of age-period-cohort methodology, but future studies could explore alternative approaches for model validation. Furthermore, the biological mechanisms (*e.g*., sex hormone-related pathways, gut microbiota modulation) underlying the observed sex disparities were not explored in this analysis and remain an important area for future investigation.

However, one major challenge should be recognized. Most studies identified the causal roles of risk factors for gastrointestinal cancers; however they included both young participants (< 50 years) and participants aged ≥ 50 years, or they recruited only young participants but analyzed the risk factor exposures during adulthood rather than early-life (*i.e*., prenatal to adolescent life). Further prospective cohort studies, especially life-course cohort studies, are therefore needed to evaluate possible risk factors of gastrointestinal cancers in China, especially early-life risk factor exposure in young populations aged < 50 years.

## Conclusions

This study highlighted the efforts made by China's government to confront its rapidly increasing burden of gastrointestinal cancers, leading to positive results in reducing the incidences of esophageal, gastric, and liver cancers. However, challenges still exist, including the increasing trends and period effects on the incidence risks of early-onset colorectal and pancreatic cancers. These results provided helpful information to support the early detection, effective prevention, and future public health policies, and demonstrated the importance of healthy lifestyles, including enough exercise, a healthy diet, no smoking, and no alcohol among the younger population, to achieve the aims of “Healthy China Action”. Furthermore, the pronounced and persistent male–female disparities in incidence and mortality underscore the critical need for sex-tailored prevention and early detection strategies.

## Supplementary Material

Supplementary Material Details
