# Supplementary material for: Sex differences in gastrointestinal cancers incidence and mortality among adolescents and young adults in China: an age-period-birth cohort study
Source: J Transl Int Med. 2026 Aug 12;14(5):719–35. doi: 10.1515/jtim-2026-0028 (PMC13726669; doi:10.1515/jtim-2026-0028)
Supplement: Supplementary file 1 [file jtim-2026-0028_sm.pdf]

Supplementary materials

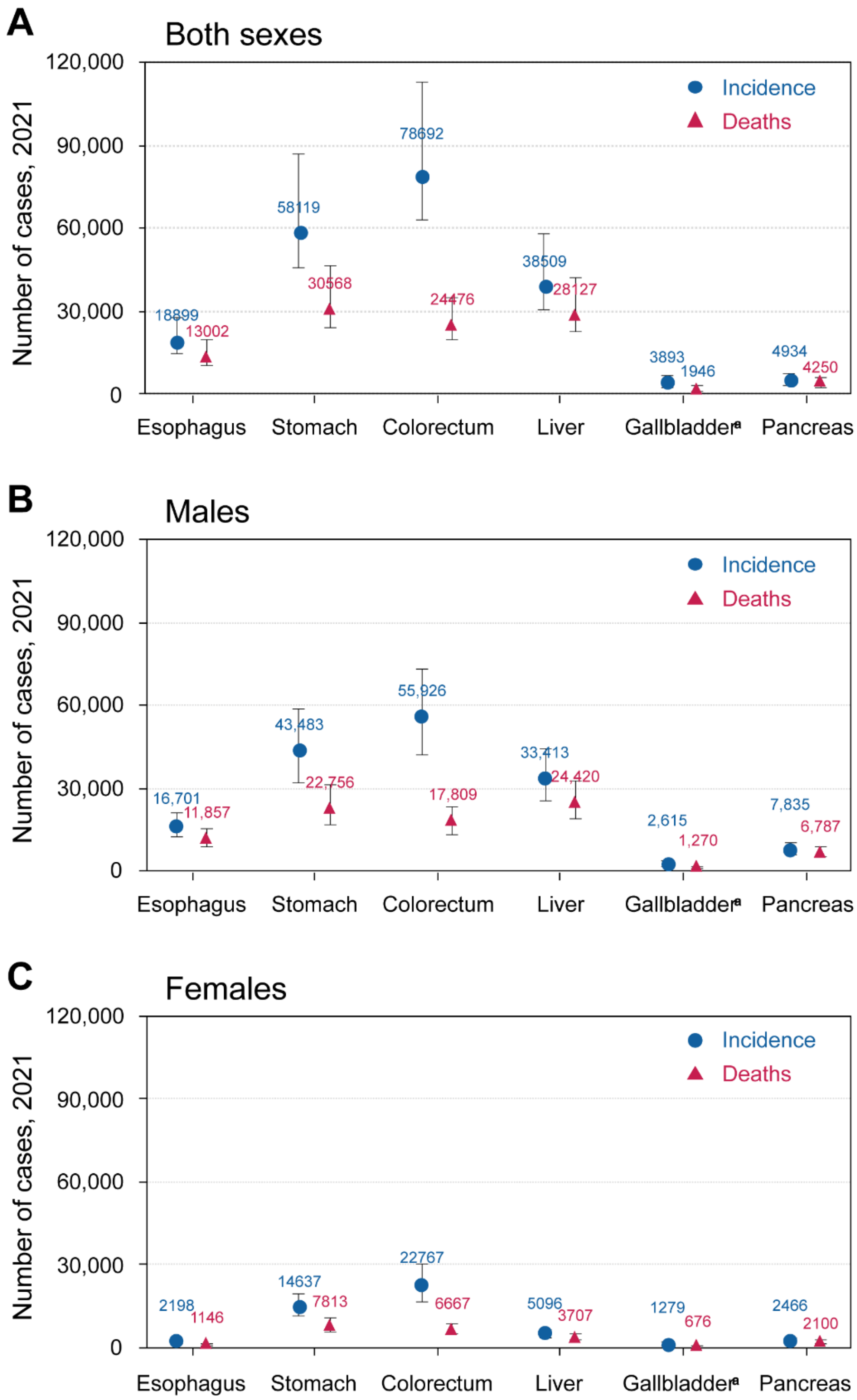

Supplementary Figure S1: Incidences and deaths for early-onset gastrointestinal cancers, 2021. (A) Both sexes, (B) males, (C) females. [a]Gallbladder and biliary tract cancers.

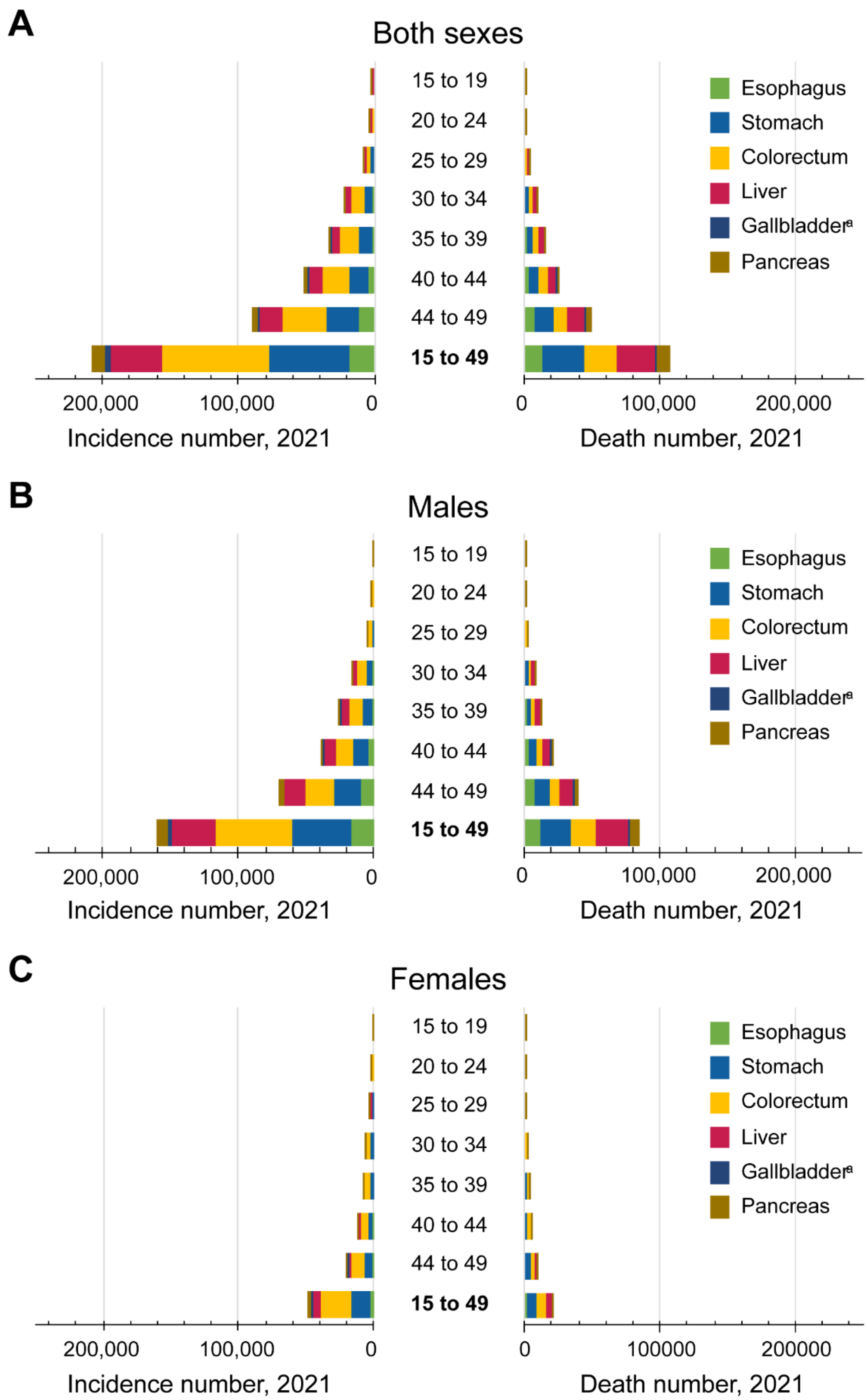

Supplementary Figure S2: Incidences and deaths for early-onset gastrointestinal cancers in relation to age, 2021. (A) Both sexes, (B) males, (C) females. [a]Gallbladder and biliary tract cancers.

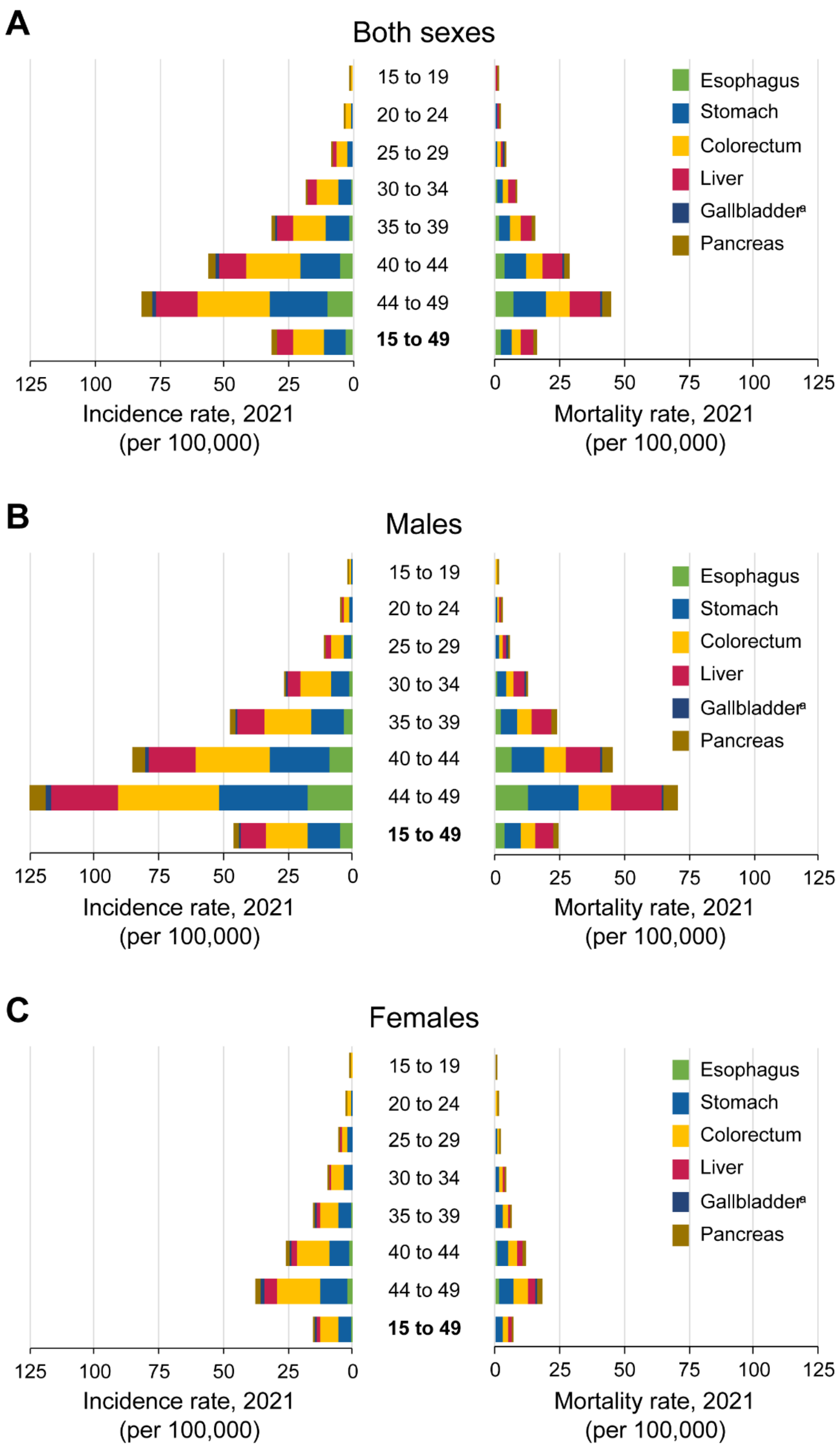

Supplementary Figure S3: Incidence and mortality rates of early-onset gastrointestinal cancers in relation to age, 2021. (A) Both sexes, (B) males, (C) females. [a]Gallbladder and biliary tract cancers.

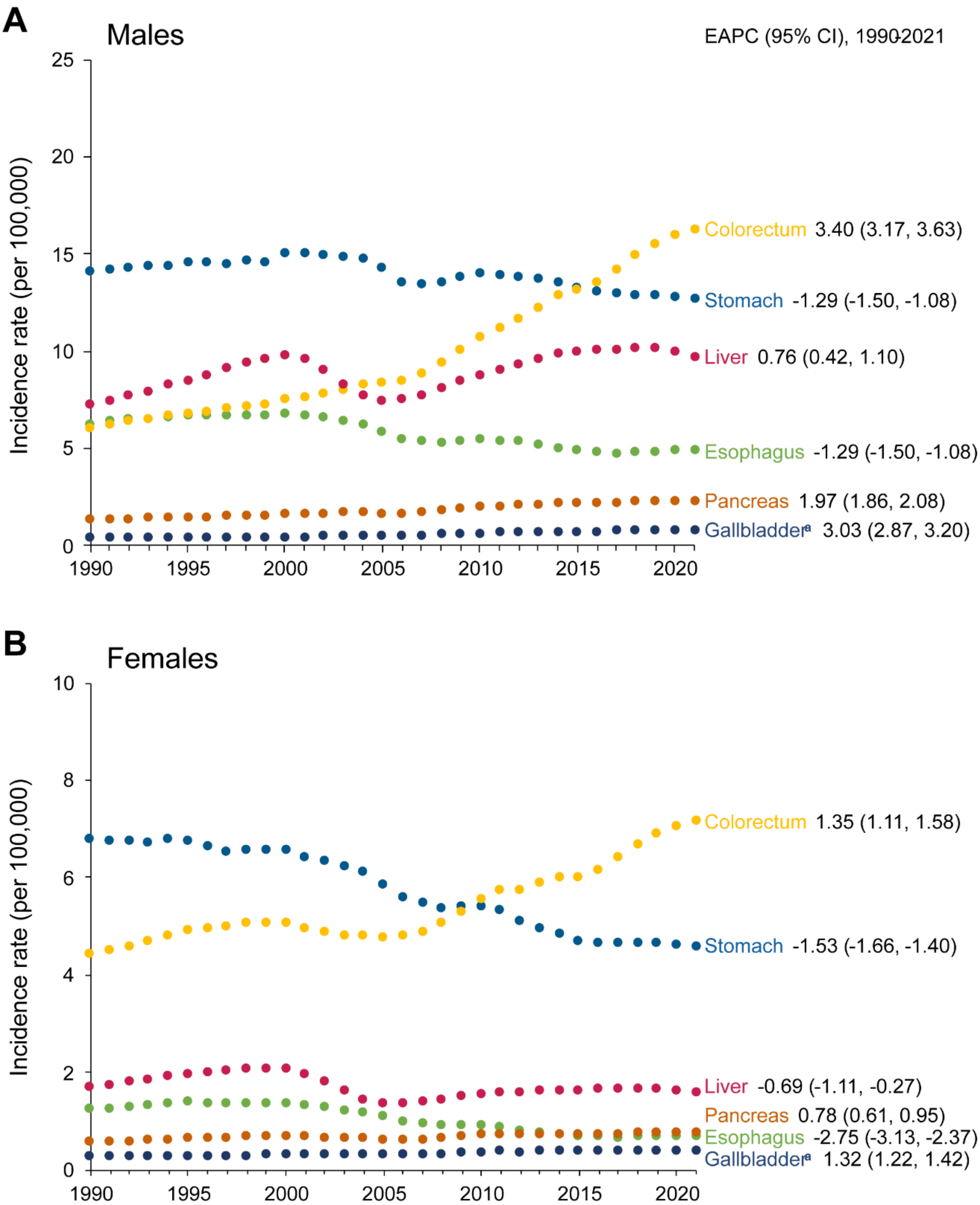

Supplementary Figure S4: Trends in incidence rates of early-onset gastrointestinal cancers, 1990–2021. (A) Males, (B) females. [a]Gallbladder and biliary tract cancers; EAPC, estimated annual percentage changes. EAPC with 95% confidence intervals (CIs) were analyzed for incidence and mortality rates by Joinpoint regression.

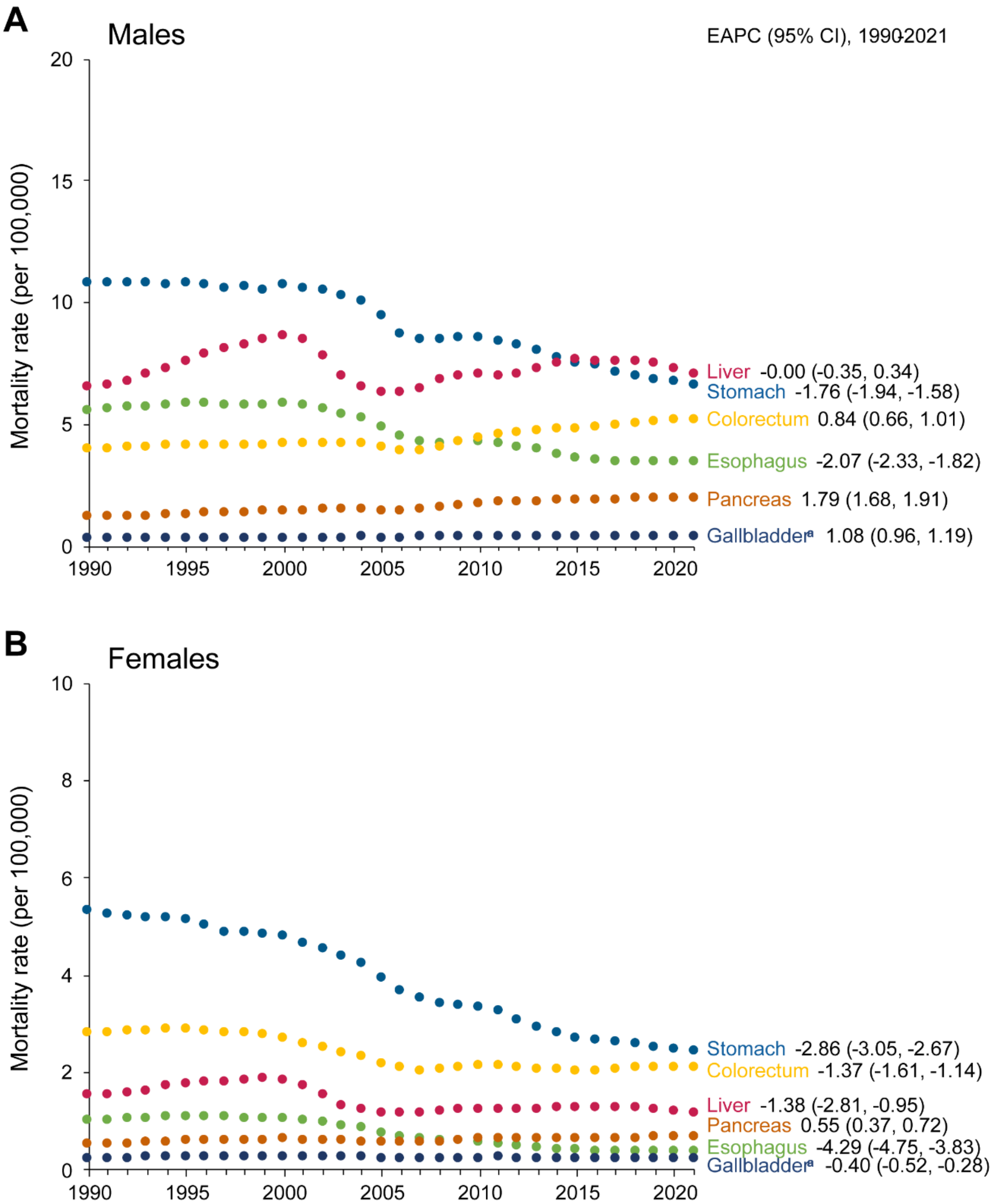

Supplementary Figure S5: Trends in mortality rates of early-onset gastrointestinal cancers, 1990−2021. (A) Males, (B) females. [a]Gallbladder and biliary tract cancers; EAPC, estimated annual percentage changes. EAPC with 95% confidence intervals (CIs) were analyzed for incidence and mortality rates by Joinpoint regression.

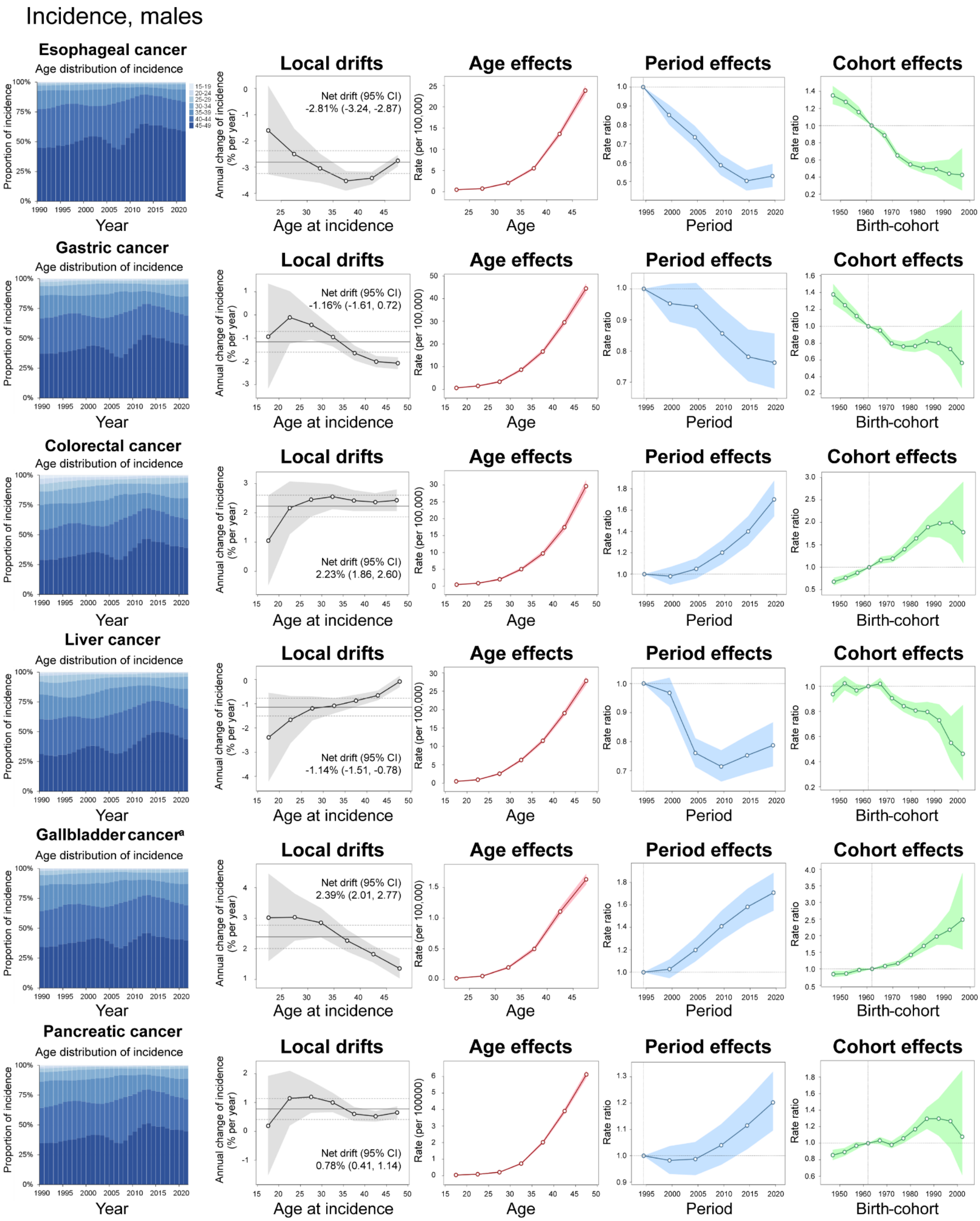

Supplementary Figure S6: Age-period-cohort analysis of incidences of early-onset gastrointestinal cancers in males. Age distribution of incidence shows relative proportion of incidence in each age group during 1990–2021. Local drift indicates annual percentage change in incidence (% per year) across the 5-year age groups 15

–19 (first dot) to 45–49 (last dot) years, and net drift (horizontal line) indicates annual percentage change in incidence (% per year) for the whole population. Age effects represented by fitted longitudinal age curves of incidence adjusted for period deviations, and Y-axis represents expected age-specific rates (per 100,000) adjusted for period effects. Period effects represented by relative risk of incidence (incidence rate ratio) and computed as ratio of age-specific rates in each period compared with the reference period (1992–1996, first dot). Birth-cohort effects represented by relative risk of incidence (incidence rate ratio) and computed as ratio of age-specific rates in each cohort compared with the reference birth cohort (1960–1964, fourth dot). Shaded areas indicate corresponding 95% confidence intervals (CIs) of each point estimate. [a]Gallbladder and biliary tract cancers. For esophageal cancer and gallbladder and biliary tract cancer populations, we only included patients aged 20–49 years because no incidence or mortality data were available for 15–19-year-olds in the GBD 2021 study.

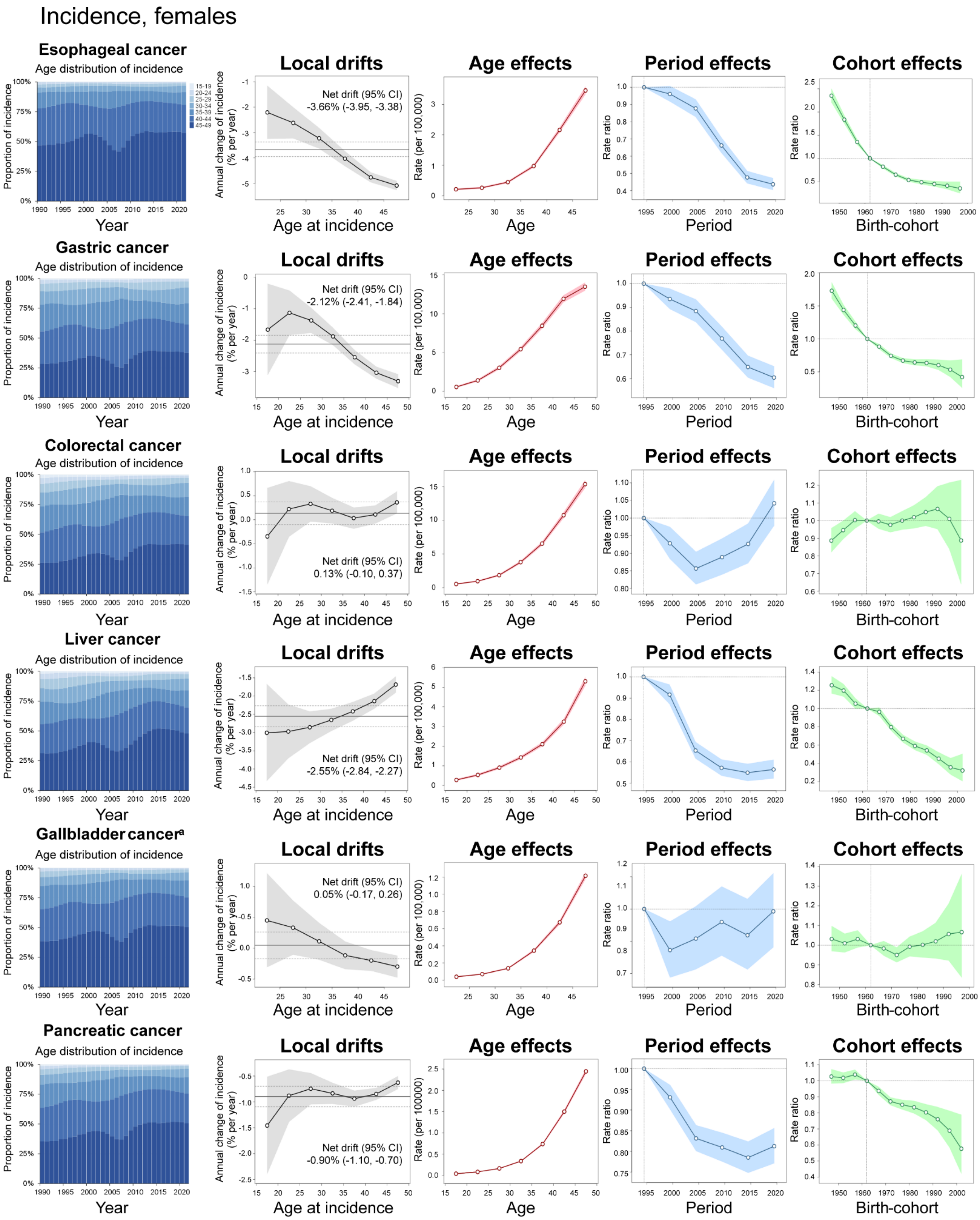

Supplementary Figure S7: Age-period-cohort analysis of incidences of early-onset gastrointestinal cancers in females. Age distribution of incidence shows relative proportion of incidence in each age group during 1990–2021. Local drift indicates annual percentage change in incidence (% per year) across the 5-year age groups 15

−19 (first dot) to 45−49 (last dot) years, and net drift (horizontal line) indicates annual percentage change in incidence (% per year) for the whole population. Age effects represented by fitted longitudinal age curves of incidence adjusted for period deviations, and Y-axis represents expected age-specific rates (per 100,000) adjusted for period effects. Period effects represented by relative risk of incidence (incidence rate ratio) and computed as ratio of age-specific rates in each period compared with the reference period (1992−1996, first dot). Birth-cohort effects represented by relative risk of incidence (incidence rate ratio) and computed as ratio of age-specific rates in each cohort compared with the reference birth cohort (1960−1964, fourth dot). Shaded areas indicate corresponding 95% confidence intervals (CIs) of each point estimate. [a] Gallbladder and biliary tract cancers. For esophageal cancer and gallbladder and biliary tract cancer populations, we only included patients aged 20−49 years because no incidence or mortality data were available for 15−19-year-olds in the GBD 2021 study.

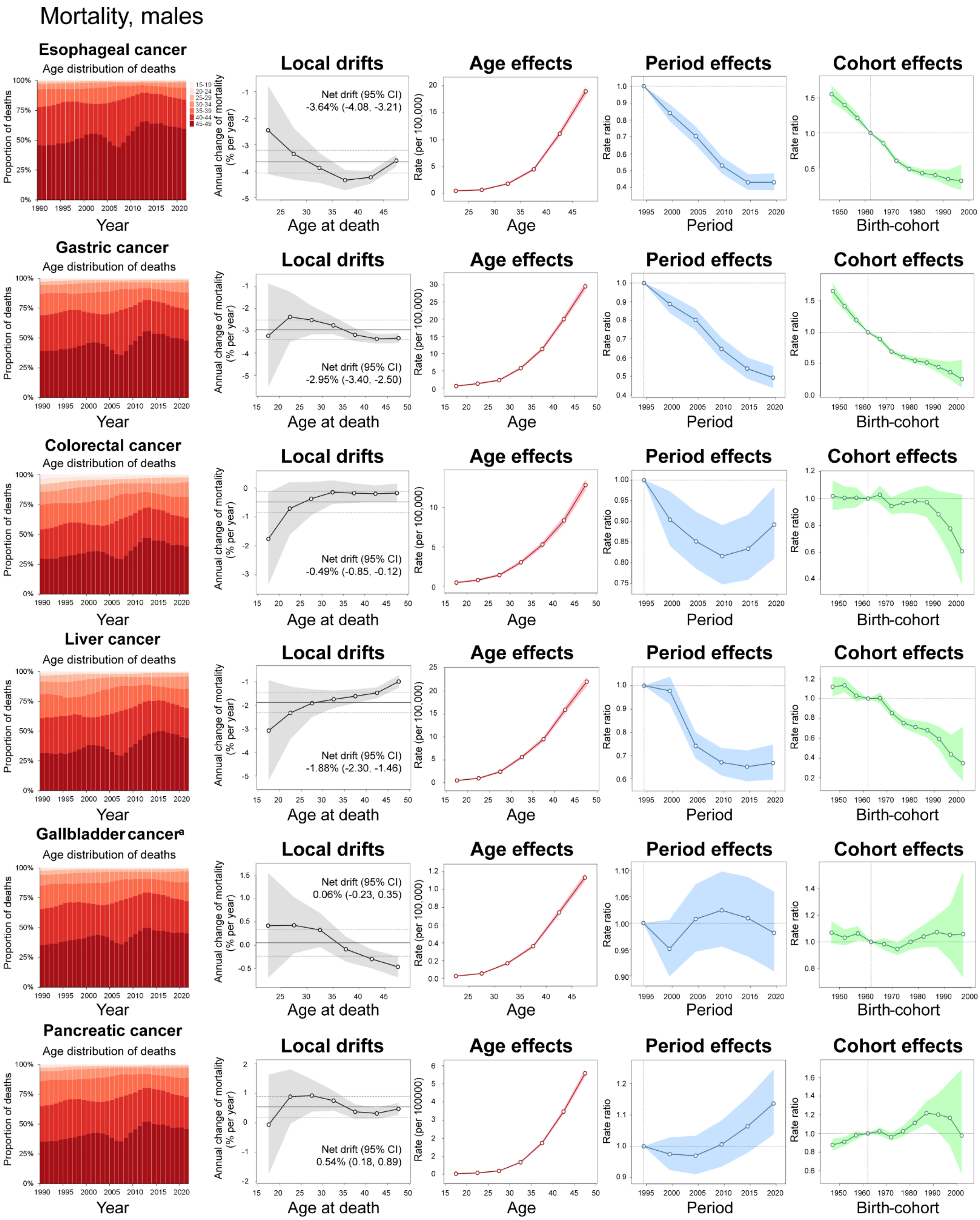

Supplementary Figure S8: Age-period-cohort analysis of mortality for early-onset gastrointestinal cancers in males. Age distribution of mortality shows relative proportion of deaths in each age group during 1990−2021.

Local drift indicates annual percentage change in mortality (% per year) across the 5-year age groups 15−19 (first dot) to 45−49 (last dot) years, and net drift (horizontal line) indicates annual percentage change in mortality (% per year) for the whole population. Age effects represented by fitted longitudinal age curves of mortality adjusted for period deviations, and Y-axis represents expected age-specific rates (per 100,000) adjusted for period effects. Period effects represented by relative risk of mortality (mortality rate ratio) and computed as ratio of age-specific rates in each period compared with the reference period (1992−1996, first dot). Birth-cohort effects represented by relative risk of mortality (mortality rate ratio) and computed as ratio of age-specific rates in each cohort compared with the reference birth cohort (1960−1964, fourth dot). Shaded areas indicate corresponding 95% confidence intervals (CIs) of each point estimate. [a] Gallbladder and biliary tract cancers. For esophageal cancer and gallbladder and biliary tract cancer populations, we only included patients aged 20−49 years because no incidence or mortality data were available for 15−19-year-olds in the GBD 2021 study.

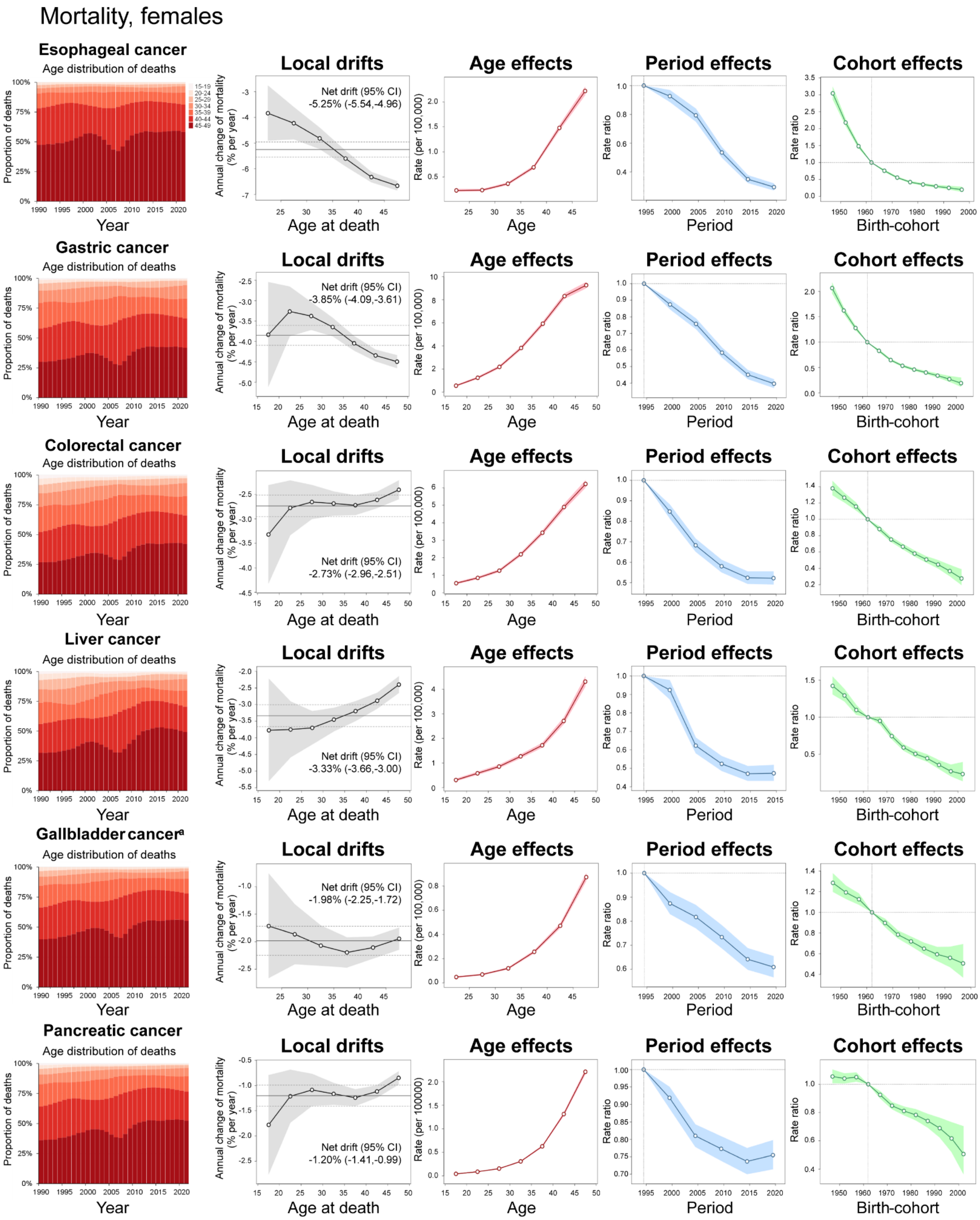

Supplementary Figure S9: Age-period-cohort analysis of mortality for early-onset gastrointestinal cancers in females. Age distribution of mortality shows relative proportion of deaths in each age group during 1990–

2021. Local drift indicates annual percentage change in mortality (% per year) across the 5-year age groups 15–19 (first dot) to 45–49 (last dot) years, and net drift (horizontal line) indicates annual percentage change in mortality (% per year) for the whole population. Age effects represented by fitted longitudinal age curves of mortality adjusted for period deviations, and Y-axis represents expected age-specific rates (per 100,000) adjusted for period effects. Period effects represented by relative risk of mortality (mortality rate ratio) and computed as ratio of age-specific rates in each period compared with the reference period (1992–1996, first dot). Birth-cohort effects represented by relative risk of mortality (mortality rate ratio) and computed as ratio of age-specific rates in each cohort compared with the reference birth cohort (1960–1964, fourth dot). Shaded areas indicate corresponding 95% confidence intervals (CIs) of each point estimate. [a] Gallbladder and biliary tract cancers. For esophageal cancer and gallbladder and biliary tract cancer populations, we only included patients aged 20–49 years because no incidence or mortality data were available for 15–19-year-olds in the GBD 2021 study.

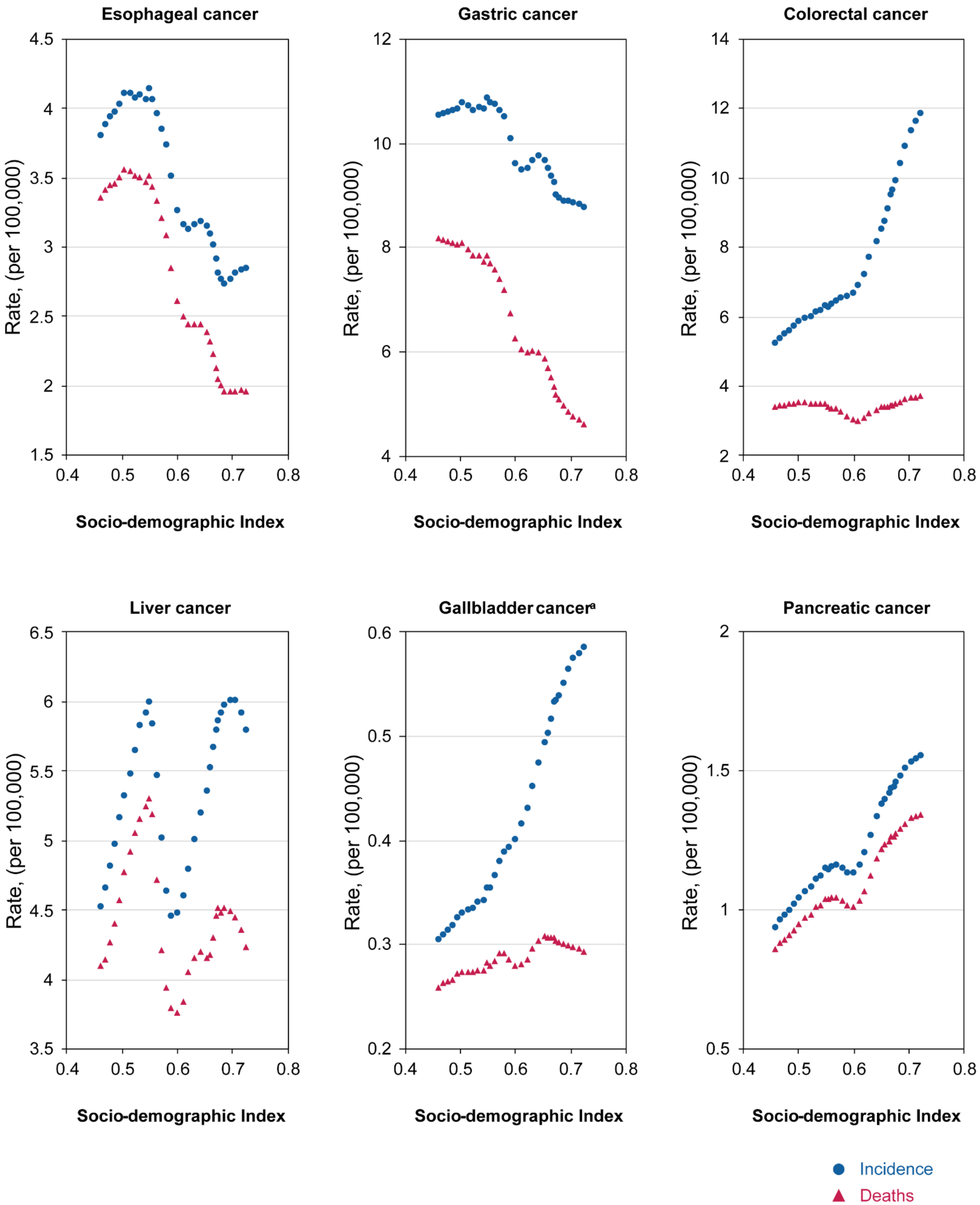

Supplementary Figure S10: Correlation between Socio-demographic Index and incidence and death rates of early-onset gastrointestinal cancers in China, 1990–2021. [a]Gallbladder and biliary tract cancers; SDI: Socio-demographic Index.

Supplementary Table S1: Incidence of early-onset gastrointestinal cancers, 1990−2021

Cancer site	1990<br>Incident cases<br>Number (95% UI)	Male/female	Incidence rate[b]<br>(95% UI)	2021<br>Incident cases<br>Number (95% UI)	Male/female	Incidence rate[b]<br>(95% UI)
Esophagus	25380<br>(21029, 30165)	5.38	3.81<br>(3.15, 4.52)	18899<br>(14640, 23589)	7.60	2.85<br>(2.21, 3.56)
Males	21401<br>(17591, 26342)	NA	6.21<br>(5.10, 7.64)	16701<br>(12524, 21370)	NA	4.85<br>(3.63, 6.20)
Females	3979<br>(1854, 5183)	NA	1.23<br>(0.58, 1.61)	2198<br>(1579, 3003)	NA	0.69<br>(0.50, 0.94)
Stomach	70248<br>(55606, 82397)	2.20	10.53<br>(8.34, 12.36)	58119<br>(45912, 74438)	2.97	8.76<br>(6.92, 11.22)
Males	48330<br>(34297, 59090)	NA	14.02<br>(9.95, 17.15)	43483<br>(32129, 58721)	NA	12.62<br>(9.32, 17.04)
Females	21919<br>(19061, 27782)	NA	6.80<br>(5.29, 8.62)	14637<br>(11187, 19676)	NA	4.59<br>(3.51, 6.17)
Colorectum	35027<br>(29409, 40729)	1.46	5.25<br>(4.41, 6.11)	78692<br>(62703, 96468)	2.46	11.86<br>(9.45, 14.54)
Males	20797<br>(15728, 25357)	NA	6.03<br>(4.56, 7.36)	55926<br>(41917, 72753)	NA	16.23<br>(12.16, 21.11)
Females	14230<br>(11034, 18060)	NA	4.42<br>(3.42, 5.61)	22767<br>(16569, 30498)	NA	7.14<br>(5.20, 9.57)
Liver	30185<br>(24839, 36227)	4.51	4.53<br>(3.72, 5.43)	38509<br>(30544, 50232)	6.56	5.81<br>(4.60, 7.57)
Males	24710<br>(19819, 30427)	NA	7.17<br>(5.75, 8.83)	33413<br>(25384, 44102)	NA	9.70<br>(7.37, 12.80)
Females	5475<br>(4250, 6857)	NA	1.70<br>(1.32, 2.13)	5096<br>(3789, 6742)	NA	1.61<br>(1.19, 2.12)
Gallbladder[a]	2035<br>(1359, 2460)	1.33	0.31<br>(0.20, 0.37)	3893<br>(2417, 5089)	2.04	0.59<br>(0.36, 0.77)
Males	1161<br>(711, 1523)	NA	0.34<br>(0.21, 0.44)	2615<br>(1414, 3657)	NA	0.76<br>(0.41, 1.06)
Females	874<br>(513, 1181)	NA	0.27<br>(0.16, 0.37)	1279<br>(799, 1824)	NA	0.40<br>(0.25, 0.57)
Pancreas	6241<br>(5256, 7386)	2.39	0.94<br>(0.79, 1.11)	10302<br>(7944, 12878)	3.18	1.55<br>(1.20, 1.94)
Males	4402<br>(3613, 5328)	NA	1.28<br>(1.05, 1.55)	7835<br>(5936, 10190)	NA	2.27<br>(1.72, 2.96)
Females	1839<br>(1395, 2355)	NA	0.57<br>(0.43, 0.73)	2466<br>(1785, 3278)	NA	0.77<br>(0.56, 1.03)

NA, not available; UI, uncertainty interval. [a]Gallbladder and biliary tract. [b]Per 100,000.

Supplementary Table S2: Mortality of early-onset gastrointestinal cancers, 1990−2021

	1990			2021		
	Number of deaths		Mortality rate[b]	Number of deaths		Mortality rate[b]
**Cancer site**	**Number (95% UI)**	**Male/female**	**(95% UI)**	**Number (95% UI)**	**Male/female**	**(95% UI)**
Esophagus	22353 (18486, 26567)	5.85	3.35 (2.77, 3.98)	13002 (10030, 16498)	10.35	1.96 (1.51, 2.49)
Males	19090 (15705, 23532)	NA	5.54 (4.56, 6.83)	11857 (8859, 15288)	NA	3.44 (2.57, 4.44)
Females	3263 (1539, 4246)	NA	1.01 (0.48, 1.32)	1146 (839, 1570)	NA	0.36 (0.26, 0.49)
Stomach	54445 (43553, 63914)	2.18	8.16 (6.53, 9.58)	30568 (23988, 39506)	2.91	4.61 (3.62, 5.96)
Males	37301 (26399, 45627)	NA	10.82 (7.66, 13.24)	22756 (16744, 30877)	NA	6.60 (4.86, 8.96)
Females	17145 (13373, 21683)	NA	5.32 (4.15, 6.73)	7813 (5919, 10443)	NA	2.45 (1.86, 3.28)
Colorectum	22619 (18986, 26252)	1.51	3.39 (2.85, 3.94)	24476 (19469, 29983)	2.67	3.69 (2.94, 4.52)
Males	13597 (10319, 16610)	NA	3.95 (2.99, 4.82)	17809 (13403, 23235)	NA	5.17 (3.89, 6.74)
Females	9022 (6988, 11475)	NA	2.80 (2.17, 3.56)	6667 (4832, 8938)	NA	2.09 (1.52, 2.80)
Liver	27360 (22538, 32769)	4.50	4.10 (3.38, 4.91)	28127 (22351, 36519)	6.59	4.24 (3.37, 5.51)
Males	22389 (17971, 27511)	NA	6.50 (5.21, 7.98)	24420 (18516, 32241)	NA	7.09 (5.37, 9.39)
Females	4971 (3868, 6195)	NA	1.54 (1.20, 1.92)	3707 (2741, 4845)	NA	1.16 (0.86, 1.52)
Gallbladder[a]	1731 (1159, 2090)	1.30	0.26 (0.17, 0.31)	1946 (1260, 2534)	1.88	0.29 (0.19, 0.38)
Males	978 (599, 1290)	NA	0.28 (0.17, 0.37)	1270 (713, 1784)	NA	0.37 (0.21, 0.52)
Females	753 (440, 1018)	NA	0.23 (0.14, 0.32)	676 (428, 975)	NA	0.21 (0.13, 0.31)
Pancreas	5687 (4792, 6711)	2.40	0.85 (0.72, 1.01)	8887 (6862, 11112)	3.23	1.34 (1.03, 1.68)
Males	4015 (3308, 4870)	NA	1.17 (0.96, 1.41)	6787 (5145, 8853)	NA	1.97 (1.49, 2.57)
Females	1672 (1269, 2135)	NA	0.52 (0.39, 0.66)	2100 (1508, 2778)	NA	0.66 (0.47, 0.87)

NA, not available; UI, uncertainty interval. [a]Gallbladder and biliary tract. [b]Per 100,000.

Supplementary Table S3: Incidence and mortality of early-onset esophageal cancer in relation to age, 1990–2021

	1990			2021		
Age	Number of cases		Rate (per 100,000)	Number of cases		Rate (per 100,000)
Incidence	Number (95% UI)	Male/female	(95% UI)	Number (95% UI)	Male/female	(95% UI)
15-19 years	NA	NA	NA	NA	NA	NA
20-24 years	315	2.80	0.24	120	3.64	0.16
	(259, 367)		(0.20, 0.28)	(95, 151)		(0.13, 0.21)
25-29 years	444	3.38	0.40	218	4.11	0.25
	(375, 511)		(0.34, 0.46)	(178, 264)		(0.21, 0.31)
30-34 years	1131	5.60	1.28	864	6.35	0.71
	(970, 1319)		(1.10, 1.49)	(705, 1048)		(0.58, 0.86)
35-39 years	3848	6.24	4.21	1976	7.28	1.86
	(3206, 4623)		(3.51, 5.06)	(1574, 2483)		(1.49, 2.34)
40-44 years	8144	5.60	12.14	4646	8.05	5.08
	(6608, 9849)		(9.85, 14.68)	(3650, 5859)		(3.99, 6.40)
45-49 years	11498	5.19	22.27	11076	7.79	10.04
	(9392, 13819)		(18.19, 26.77)	(8442, 14216)		(7.65, 12.89)
Mortality						
15-19 years	NA	NA	NA	NA	NA	NA
20-24 years	307	3.05	0.23	89	4.96	0.12
	(254, 359)		(0.19, 0.27)	(70, 114)		(0.10, 0.16)
25-29 years	396	3.67	0.36	149	5.64	0.17
	(335, 458)		(0.31, 0.42)	(120, 179)		(0.14, 0.21)
30-34 years	1000	6.10	1.13	588	8.76	0.49
	(856, 1171)		(0.97, 1.33)	(475, 715)		(0.39, 0.59)
35-39 years	3225	6.81	3.53	1282	10.01	1.21
	(2682, 3866)		(2.94, 4.23)	(1010, 1621)		(0.95, 1.53)
40-44 years	7128	6.12	10.62	3166	11.04	3.46
	(5801, 8638)		(8.65, 12.87)	(2444, 4002)		(2.67, 4.37)
45-49 years	10297	5.65	19.95	7729	10.56	7.01
	(8463, 12383)		(16.39, 23.99)	(5839, 10098)		(5.29, 9.15)

NA, not available; UI, uncertainty interval.

Supplementary Table S4: Incidence and mortality of early-onset gastric cancer in relation to age, 1990−2021

	1990			2021		
Age	Number of cases		Rate (per 100,000)	Number of cases		Rate (per 100,000)
Incidence	Number (95% UI)	Male/female	(95% UI)	Number (95% UI)	Male/female	(95% UI)
15-19 years	708	1.49	0.56	222	1.71	0.30
	(582, 853)		(0.46, 0.67)	(172, 276)		(0.23, 0.37)
20-24 years	1653	1.33	1.25	723	1.73	0.99
	(1381, 1940)		(1.05, 1.47)	(561, 887)		(0.77, 1.21)
25-29 years	3001	1.17	2.73	2078	1.77	2.40
	(2513, 3541)		(2.29, 3.22)	(1589, 2489)		(1.84, 2.88)
30-34 years	6326	1.59	7.17	6555	2.33	5.41
	(5215, 7418)		(5.91, 8.41)	(5066, 8039)		(4.18, 6.64)
35-39 years	14100	1.86	15.44	9449	2.65	8.92
	(11315, 16500)		(12.39, 18.06)	(7437, 11769)		(7.02, 11.11)
40-44 years	20250	2.38	30.18	14413	3.11	15.75
	(15840, 23726)		(23.61, 35.36)	(11252, 18592)		(12.29, 20.31)
45-49 years	24211	2.91	46.90	24679	3.50	22.37
	(18687, 28869)		(36.20, 55.93)	(18909, 32465)		(17.14, 29.43)
Mortality						
15-19 years	517	1.42	0.41	92	1.60	0.12
	(427, 617)		(0.34, 0.49)	(71, 114)		(0.10, 0.15)
20-24 years	1249	1.26	0.95	305	1.61	0.42
	(1058, 1467)		(0.80, 1.11)	(240, 373)		(0.33, 0.51)
25-29 years	2064	1.11	1.88	791	1.66	0.92
	(1733, 2444)		(1.58, 2.22)	(622, 960)		(0.72, 1.11)
30-34 years	4501	1.53	5.10	2754	2.20	2.27
	(3709, 5279)		(4.20, 5.98)	(2126, 3388)		(1.75, 2.80)
35-39 years	10399	1.81	11.38	4650	2.55	4.39
	(8434, 12129)		(9.23, 13.28)	(3634, 5794)		(3.43, 5.47)
40-44 years	15900	2.33	23.70	7746	3.01	8.46
	(12436, 18630)		(18.54, 27.77)	(6087, 9904)		(6.65, 10.92)
45-49 years	19816	2.85	38.39	14230	3.36	12.90
	(15351, 23725)		(29.74, 45.96)	(10925, 18684)		(9.90, 16.94)

UI: uncertainty interval.

Supplementary Table S5: Incidence and mortality of early-onset colorectal cancer in relation to age, 1990–2021

	1990			2021		
Age	Number of cases		Rate (per 100,000)	Number of cases		Rate (per 100,000)
Incidence	Number (95% UI)	Male/female	(95% UI)	Number (95% UI)	Male/female	(95% UI)
15-19 years	1038	1.52	0.82	563	2.05	0.75
	(820, 1220)		(0.65, 0.96)	(444, 703)		(0.59, 0.94)
20-24 years	1608	1.34	1.22	1233	2.17	1.69
	(1296, 1909)		(0.98, 1.45)	(983, 1513)		(1.34, 2.07)
25-29 years	2303	1.28	2.10	3273	2.40	3.78
	(1859, 2728)		(1.69, 2.48)	(2685, 3969)		(3.10, 4.59)
30-34 years	4033	1.42	4.57	9823	2.68	8.11
	(3300, 4680)		(3.74, 5.30)	(7983, 11973)		(6.59, 9.88)
35-39 years	7460	1.40	8.17	13623	2.67	12.86
	(6242, 8735)		(6.83, 9.56)	(10992, 16811)		(10.37, 15.86)
40-44 years	8857	1.43	13.20	19007	2.46	20.77
	(7314, 10412)		(10.90, 15.52)	(15031, 23547)		(16.42, 25.73)
45-49 years	9729	1.63	18.85	31169	2.33	28.25
	(8089, 11387)		(15.67, 22.06)	(24185, 38761)		(21.92, 35.13)
Mortality						
15-19 years	710	1.58	0.56	183	2.28	0.25
	(558, 840)		(0.44, 0.66)	(142, 229)		(0.19, 0.31)
20-24 years	1132	1.38	0.86	411	2.39	0.56
	(913, 1345)		(0.69, 1.02)	(326, 504)		(0.45, 0.69)
25-29 years	1467	1.32	1.34	982	2.66	1.14
	(1180, 1736)		(1.07, 1.58)	(804, 1192)		(0.93, 1.38)
30-34 years	2518	1.47	2.85	2888	2.97	2.38
	(2050, 2919)		(2.32, 3.31)	(2343, 3524)		(1.93, 2.91)
35-39 years	4676	1.45	5.12	4223	2.89	3.99
	(3895, 5472)		(4.26, 5.99)	(3383, 5209)		(3.19, 4.92)
40-44 years	5665	1.47	8.44	5833	2.66	6.37
	(4694, 6637)		(7.00, 9.89)	(4558, 7318)		(4.98, 8.00)
45-49 years	6452	1.67	12.50	9955	2.53	9.02
	(5343, 7553)		(10.35, 14.63)	(7739, 12523)		(7.02, 11.35)

UI: uncertainty interval.

Supplementary Table S6: Incidence and mortality of early-onset liver cancer in relation to age, 1990−2021

	1990			2021		
Age	Number of cases		Rate (per 100,000)	Number of cases		Rate (per 100,000)
Incidence	Number (95% UI)	Male/female	(95% UI)	Number (95% UI)	Male/female	(95% UI)
15-19 years	349	2.35	0.28	130	2.63	0.17
	(290, 427)		(0.23, 0.34)	(105, 167)		(0.14, 0.22)
20-24 years	790	2.11	0.60	308	2.92	0.42
	(646, 968)		(0.49, 0.73)	(224, 409)		(0.31, 0.56)
25-29 years	1623	2.86	1.48	1175	4.99	1.36
	(1335, 2000)		(1.21, 1.82)	(936, 1458)		(1.08, 1.69)
30-34 years	3243	4.30	3.68	3880	6.83	3.20
	(2674, 3891)		(3.03, 4.41)	(3037, 5021)		(2.51, 4.14)
35-39 years	6170	4.99	6.76	6368	7.68	6.01
	(5074, 7525)		(5.56, 8.24)	(4849, 8334)		(4.58, 7.87)
40-44 years	8586	5.38	12.80	9556	7.56	10.44
	(6933, 10545)		(10.33, 15.72)	(7306, 12537)		(7.98, 13.70)
45-49 years	9424	4.51	18.26	17092	6.00	15.49
	(7528, 11470)		(14.58, 22.22)	(12848, 22303)		(11.65, 20.22)
Mortality						
15-19 years	340	2.36	0.27	103	2.72	0.14
	(282, 417)		(0.22, 0.35)	(83, 133)		(0.11, 0.18)
20-24 years	799	2.12	0.61	254	3.00	0.35
	(654, 974)		(0.50, 0.74)	(183, 339)		(0.25, 0.46)
25-29 years	1500	2.88	1.37	885	5.17	1.02
	(1235, 1850)		(1.12, 1.68)	(705, 1104)		(0.82, 1.28)
30-34 years	2956	4.33	3.35	2895	7.14	2.39
	(2442, 3583)		(2.77, 4.06)	(2267, 3736)		(1.87, 3.08)
35-39 years	5335	5.01	5.84	4516	8.00	4.26
	(4394, 6482)		(4.81, 7.10)	(3428, 5903)		(3.24, 5.57)
40-44 years	7740	5.40	11.54	6891	7.70	7.53
	(6231, 9496)		(9.29, 14.15)	(5270, 9107)		(5.76, 9.95)
45-49 years	8690	4.51	16.83	12583	5.90	11.41
	(6933, 10581)		(13.43, 20.50)	(9471, 16473)		(8.59, 14.93)

UI: uncertainty interval.

Supplementary Table S7: Incidence and mortality of early-onset gallbladder/biliary tract cancer in relation to age, 1990−2021

	1990			2021		
**Age**	**Number of cases**		**Rate (per 100,000)**	**Number of cases**		**Rate (per 100,000)**
**Incidence**	**Number (95% UI)**	**Male/female**	**(95% UI)**	**Number (95% UI)**	**Male/female**	**(95% UI)**
15-19 years	NA	NA	NA	NA	NA	NA
20-24 years	49	0.84	0.04	35	1.35	0.05
	(29, 61)		(0.02, 0.05)	(23, 46)		(0.03, 0.06)
25-29 years	81	1.01	0.07	90	1.87	0.10
	(47, 98)		(0.04, 0.09)	(56, 117)		(0.06, 0.14)
30-34 years	170	1.59	0.19	344	2.94	0.28
	(104, 206)		(0.12, 0.23)	(212, 444)		(0.18, 0.37)
35-39 years	414	1.45	0.45	650	2.57	0.61
	(270, 506)		(0.30, 0.55)	(399, 849)		(0.38, 0.80)
40-44 years	586	1.49	0.87	1093	2.41	1.19
	(402, 710)		(0.60, 1.06)	(657, 1426)		(0.72, 1.56)
45-49 years	735	1.18	1.42	1682	1.62	1.52
	(515, 897)		(1.00, 1.74)	(1113, 2253)		(1.01, 2.04)
Mortality						
15-19 years	NA	NA	NA	NA	NA	NA
20-24 years	47	0.82	0.04	18	1.23	0.02
	(28, 58)		(0.02, 0.04)	(12, 24)		(0.02, 0.03)
25-29 years	69	0.99	0.06	43	1.73	005
	(41, 85)		(0.04, 0.08)	(28, 55)		(0.03, 0.06)
30-34 years	143	1.55	0.16	158	2.73	0.13
	(88, 174)		(0.10, 0.20)	(100, 203)		(0.08, 0.17)
35-39 years	331	1.42	0.36	282	2.40	0.27
	(216, 406)		(0.24, 0.44)	(177, 365)		(0.17, 0.34)
40-44 years	490	1.46	0.73	499	2.25	0.54
	(337, 596)		(0.50, 0.89)	(309, 646)		(0.34, 0.71)
45-49 years	651	1.17	1.26	947	1.53	0.86
	(456, 799)		(0.88, 1.55)	(650, 1282)		(0.59, 1.16)

NA, not available; UI: uncertainty interval.

Supplementary Table S8: Incidence and mortality of early-onset pancreatic cancer in relation to age, 1990–2021

	1990			2021		
**Age**	**Number of cases**		**Rate (per 100,000)**	**Number of cases**		**Rate (per 100,000)**
**Incidence**	**Number (95% UI)**	**Male/female**	**(95% UI)**	**Number (95% UI)**	**Male/female**	**(95% UI)**
15-19 years	69	1.74	0.05	29	2.20	0.04
	(57, 81)		(0.05, 0.06)	(23, 37)		(0.03, 0.05)
20-24 years	125	1.51	0.10	72	2.22	0.10
	(102, 147)		(0.08, 0.11)	(56, 90)		(0.08, 0.12)
25-29 years	214	1.50	0.19	202	2.49	0.23
	(181, 249)		(0.17, 0.23)	(162, 248)		(0.19, 0.29)
30-34 years	510	2.34	0.58	832	3.70	0.69
	(434, 596)		(0.49, 0.68)	(669, 997)		(0.55, 0.82)
35-39 years	1316	2.70	1.44	1686	4.04	1.59
	(1104, 1536)		(1.21, 1.68)	(1321, 2093)		(1.25, 1.98)
40-44 years	1831	2.52	2.73	2729	3.44	2.98
	(1517, 2205)		(2.26, 3.29)	(2122, 3410)		(2.32, 3.73)
45-49 years	2177	2.35	4.22	4752	2.80	4.31
	(1795, 2640)		(3.48, 5.11)	(3600, 6109)		(3.26, 5.54)
Mortality						
15-19 years	67	1.76	0.05	27	2.27	0.04
	(56, 79)		(0.04, 0.06)	(21, 34)		(0.03, 0.05)
20-24 years	127	1.52	0.10	69	2.29	0.09
	(104, 149)		(0.08, 0.11)	(53, 86)		(0.07, 0.12)
25-29 years	198	1.51	0.18	175	2.57	0.20
	(168, 231)		(0.15, 0.21)	(140, 215)		(0.16, 0.25)
30-34 years	465	2.36	0.53	711	3.82	0.59
	(397, 542)		(0.45, 0.61)	(570, 856)		(0.47, 0.71)
35-39 years	1140	2.72	1.25	1367	4.16	1.29
	(956, 1335)		(1.05, 1.46)	(1070, 1698)		(1.01, 1.60)
40-44 years	1657	2.54	2.47	2312	3.54	2.53
	(1371, 1993)		(2.04, 2.97)	(1802, 2874)		(1.97, 3.14)
45-49 years	2033	2.36	3.94	4226	2.84	3.83
	(1677, 2465)		(3.25, 4.77)	(3196, 5441)		(2.90, 4.93)

UI: uncertainty interval.

Supplementary Table S9: Mortality-to-incidence ratio of early-onset gastrointestinal cancers in relation to age, 2021

	Mortality-to-incidence ratio[b] (number of death cases/number of incidence cases), 2021					
**Population**	**Esophageal cancer**	**Gastric cancer**	**Colorectal cancer**	**Liver cancer**	**Gallbladder cancer[a]**	**Pancreatic cancer**
Both sexes	0.69	0.53	0.31	0.73	0.50	0.86
15-19 years	NA	0.41	0.33	0.80	NA	0.91
20-24 years	0.74	0.42	0.33	0.82	0.53	0.95
25-29 years	0.68	0.38	0.30	0.75	0.47	0.87
30-34 years	0.68	0.42	0.29	0.75	0.46	0.86
35-39 years	0.65	0.49	0.31	0.71	0.43	0.81
40-44 years	0.68	0.54	0.31	0.72	0.46	0.85
45-49 years	0.70	0.58	0.32	0.74	0.56	0.89
Males	0.71	0.52	0.32	0.73	0.49	0.87
15-19 years	NA	0.40	0.34	0.81	NA	0.92
20-24 years	0.79	0.41	0.34	0.83	0.50	0.96
25-29 years	0.72	0.37	0.31	0.76	0.46	0.87
30-34 years	0.71	0.41	0.30	0.75	0.45	0.86
35-39 years	0.67	0.49	0.32	0.71	0.43	0.82
40-44 years	0.70	0.53	0.31	0.72	0.45	0.85
45-49 years	0.72	0.57	0.33	0.73	0.55	0.89
Females	0.52	0.53	0.29	0.73	0.53	0.85
15-19 years	NA	0.43	0.30	0.78	NA	0.90
20-24 years	0.58	0.44	0.31	0.81	0.55	0.93
25-29 years	0.53	0.40	0.28	0.73	0.50	0.85
30-34 years	0.51	0.44	0.27	0.72	0.48	0.83
35-39 years	0.49	0.51	0.29	0.68	0.46	0.79
40-44 years	0.51	0.55	0.29	0.71	0.48	0.83
45-49 years	0.53	0.59	0.30	0.75	0.58	0.88

NA, not available. [a]Gallbladder and biliary tract cancers. [b]Mortality-to-incidence ratio is calculated by the ratio between the number of death cases and the number of incidence cases.

Supplementary Table S10: Temporal trends in incidence rates for early-onset gastrointestinal cancers in relation to sex and age, 1990−2021

	Temporal trends by Joinpoint regression analysis for incidence rates (EAPC, % per year)					
	**Esophageal cancer**	**Gastric cancer**	**Colorectal cancer**	**Liver cancer**	**Gallbladder cancer[a]**	**Pancreatic cancer**
Population	EAPC (95% CI)	EAPC (95% CI)	EAPC (95% CI)	EAPC (95% CI)	EAPC (95% CI)	EAPC (95% CI)
Both sexes	-1.51 (-1.74, -1.27)	-0.75 (-0.87, -0.64)	2.67 (2.43, 2.91)	0.52 (0.16, 0.87)	2.37 (2.24, 2.51)	1.64 (1.51, 1.76)
Males	-1.29 (-1.50, -1.08)	-0.44 (-0.56, -0.31)	3.40 (3.17, 3.63)	0.76 (0.42, 1.10)	3.03 (2.87, 3.20)	1.97 (1.86, 2.08)
Females	-2.75 (-3.13, -2.37)	-1.53 (-1.66, -1.40)	1.35 (1.11, 1.58)	-0.69 (-1.11, -0.27)	1.32 (1.22, 1.42)	0.78 (0.61, 0.95)
Age group						
15-19 years	NA	-2.19 (-2.31, -2.07)	-0.62 (-1.03, -0.22)	-1.93 (-2.26, -1.60)	NA	-1.44 (-1.72, -1.16)
20-24 years	-1.65 (-1.91, -1.40)	-0.88 (-1.05, -0.72)	0.82 (0.39, 1.25)	-2.53 (-3.01, -2.05)	1.06 (0.82, 1.30)	-0.01 (-0.33, 0.31)
25-29 years	-2.11 (-2.38, -1.83)	-0.31 (-0.51, -0.12)	1.94 (1.57, 2.32)	-1.18 (-1.89, -0.47)	1.56 (1.27, 1.85)	0.65 (0.33, 0.96)
30-34 years	-2.67 (-3.06, -2.27)	-1.03 (-1.22, -0.83)	1.87 (1.53, 2.22)	-1.21 (-1.72, -0.71)	1.65 (1.32, 1.98)	0.59 (0.26, 0.92)
35-39 years	-3.74 (-4.24, -3.23)	-2.22 (-2.51, -1.92)	1.24 (0.89, 1.59)	-0.90 (-1.17, -0.62)	1.01 (0.69, 1.33)	-0.00 (-0.28, 0.27)
40-44 years	-3.68 (-4.05, -3.31)	-2.39 (-2.59, -2.18)	1.36 (1.03, 1.70)	-1.04 (-1.28, -0.80)	1.24 (0.99, 1.49)	0.14 (-0.04, 0.32)
45-49 years	-2.99 (-3.19, -2.78)	-2.37 (-2.53, -2.21)	1.53 (1.15, 1.81)	-0.42 (-0.79, 0.06)	0.43 (0.26, 0.61)	0.17 (0.02, 0.32)

CI: confidence interval; EAPC: estimated annual percentage change; NA: not available. [a]Gallbladder and biliary tract cancers.

Supplementary Table S11: Temporal trends in mortality rates for early-onset gastrointestinal cancers in relation to sex and age, 1990−2021

	Temporal trends by Joinpoint regression analysis for mortality rates (EAPC, % per year)					
	**Esophageal cancer**	**Gastric cancer**	**Colorectal cancer**	**Liver cancer**	**Gallbladder cancer[a]**	**Pancreatic cancer**
Population	EAPC (95% CI)	EAPC (95% CI)	EAPC (95% CI)	EAPC (95% CI)	EAPC (95% CI)	EAPC (95% CI)
Both sexes	-2.35 (-2.63, -2.08)	-2.08 (-2.26, -1.91)	0.08 (-0.12, 0.28)	-0.23 (-0.59, 0.13)	0.49 (0.39, 0.58)	1.45 (·.32, 1.57)
Males	-2.07 (-2.33, -1.82)	-1.76 (-1.94, -1.58)	0.84 (0.66, 1.01)	-0.00 (-0.35, 0.34)	1.08 (0.96, 1.19)	1.79 (1.68, 1.91)
Females	-4.29 (-4.75, -3.83)	-2.86 (-3.05, -2.67)	-1.37 (-1.61, -1.14)	-1.38 (-2.81, -0.95)	-0.40 (-0.52, -0.28)	0.55 (0.37, 0.72)
Age group						
15-19 years	NA	-4.13 (-4.31, -3.96)	-3.19 (-3.58, -2.79)	-2.62 (-3.03, -2.21)	NA	-1.67 (-1.95, -1.39)
20-24 years	-2.62 (-2.89, -2.34)	-2.91 (-3.09, -2.73)	-1.85 (-2.27, -1.42)	-3.12 (-3.65, -2.60)	-1.04 (-1.19, -0.89)	-0.25 (-0.56, 0.06)
25-29 years	-3.08 (-3.38, -2.77)	-2.37 (-2.55, -2.20)	-0.77 (-1.11, -0.43)	-1.83 (-2.58, -1.07)	-0.59 (-0.76, -0.41)	0.40 (0.10, 0.70)
30-34 years	-3.58 (-3.99, -3.16)	-2.85 (-3.07, -2.62)	-0.82 (-1.12, -0.52)	-1.86 (-2.43, -1.30)	-0.52 (-0.72, -0.32)	0.36 (0.04, 0.67)
35-39 years	-4.61 (-5.13, -4.09)	-3.59 (-3.91, -3.26)	-1.21 (-0.15, -0.91)	-1.50 (-1.78, -1.22)	-1.13 (-1.32, -0.93)	-0.24 (-0.51, 0.03)
40-44 years	-4.52 (-4.91, -4.13)	-3.68 (-3.90, -3.45)	-1.17 (-1.45, -0.90)	-1.82 (-2.06, -1.58)	-0.88 (-1.01, -0.76)	-0.09 (-0.26, 0.08)
45-49 years	-3.83 (-4.07, -3.59)	-3.58 (-3.76, -3.41)	-1.04 (-1.21, -0.82)	-1.25 (-1.62, -0.87)	-1.16 (-1.32, -1.01)	-0.01 (-0.14, 0.13)

CI: confidence interval; EAPC: estimated annual percentage change. NA, not available. [a]Gallbladder and biliary tract cancers.

Supplementary Table S12: Temporal trends in incidence rates for early-onset gastrointestinal cancers in relation to sex and age, 2010−2021

	Temporal trends by Joinpoint regression analysis for incidence rates (EAPC, % per year)					
	**Esophageal cancer**	**Gastric cancer**	**Colorectal cancer**	**Liver cancer**	**Gallbladder cancer[a]**	**Pancreatic cancer**
**Population**	EAPC (95% CI)	EAPC (95% CI)	EAPC (95% CI)	EAPC (95% CI)	EAPC (95% CI)	EAPC (95% CI)
Both sexes	-1.21 (-1.82, -0.61)	-1.03 (-1.24, -0.81)	3.52 (3.34, 3.71)	1.08 (0.55, 1.61)	1.86 (1.65, 2.07)	1.32 (1.19, 1.44)
Males	-1.19 (-1.71, -0.67)	-1.01 (-1.14, -0.88)	3.93 (3.72, 4.13)	1.02 (0.43, 1.61)	2.31 (1.96, 2.66)	1.39 (1.21, 1.57)
Females	-2.57 (-3.62, -1.51)	-1.44 (-1.90, -0.98)	2.38 (2.06, 2.71)	0.38 (0.04, 0.72)	0.86 (0.63, 1.09)	0.69 (0.42, 0.96)
Age group						
15-19 years	NA	-1.48 (-1.98, -0.98)	2.18 (1.93, 2.42)	-1.54 (-2.17, -0.90)	NA	0.80 (0.49, 1.12)
20-24 years	0.78 (-0.23, 1.81)	0.50 (-0.21, 1.22)	4.16 (3.55, 4.77)	0.00 (-2.16, 2.20)	2.32 (1.66, 2.99)	2.45 (1.80, 3.11)
25-29 years	-0.42 (-1.31, 0.48)	-0.34 (-0.65, -0.03)	3.45 (3.07, 3.83)	1.97 (1.12, 2.82)	1.59 (1.21, 1.97)	1.60 (1.31, 1.89)
30-34 years	0.18 (-1.32, 1.70)	-0.28 (-1.15, 0.60)	3.55 (2.89, 4.21)	0.67 (0.59, 0.74)	2.31 (1.85, 2.77)	1.74 (1.20, 2.28)
35-39 years	0.98 (-0.72, 2.71)	0.36 (-0.62, 1.35)	4.39 (3.62, 5.16)	0.45 (-0.29, 1.21)	3.40 (2.76, 4.05)	2.69 (1.95, 3.43)
40-44 years	-0.66 (-2.11, 0.80)	-0.69 (-1.45, 0.08)	3.93 (3.41, 4.44)	0.64 (0.10, 1.18)	2.50 (2.14, 2.85)	1.75 (1.33, 2.17)
45-49 years	-2.95 (-3.80, -2.10)	-3.25 (-3.75, -2.77)	1.42 (1.02, 1.83)	0.25 (-0.78, 1.30)	-0.28 (-0.68, 0.12)	-0.61 (-1.03, -0.19)

CI: confidence interval; EAPC: estimated annual percentage change; NA, not available. [a]Gallbladder and biliary tract cancers.

Supplementary Table S13: Temporal trends in mortality rates for early-onset gastrointestinal cancers in relation to sex and age, 2010−2021

	Temporal trends by Joinpoint regression analysis for mortality rates (EAPC, % per year)					
	**Esophageal cancer**	**Gastric cancer**	**Colorectal cancer**	**Liver cancer**	**Gallbladder cancer[a]**	**Pancreatic cancer**
Population	EAPC (95% CI)	EAPC (95% CI)	EAPC (95% CI)	EAPC (95% CI)	EAPC (95% CI)	EAPC (95% CI)
Both sexes	-2.16 (-2.75, -1.56)	-2.42 (-2.62, -2.22)	1.01 (0.91, 1.11)	0.42 (-0.14, 0.98)	-0.42 (-0.55, -0.29)	1.09 (0.96, 1.22)
Males	-2.11 (-2.63, -1.59)	-2.43 (-2.55, -2.31)	1.38 (1.27, 1.48)	0.37 (-0.23, 0.97)	-0.09 (-0.36, 0.19)	1.17 (0.98, 1.35)
Females	-4.16 (-5.21, -3.09)	-2.75 (-3.24, -2.26)	-0.14 (-0.48, 0.29)	-0.36 (-0.81, 0.11)	-1.13 (-1.35, -0.92)	0.45 (0.18, 0.72)
Age group						
15-19 years	NA	-3.29 (-3.80, -2.79)	-0.15 (-0.34, 0.05)	-2.24 (-2.86, -1.62)	NA	0.57 (0.24, 0.89)
20-24 years	-0.05 (-1.13, 1.05)	-1.45 (-2.21, -0.68)	1.79 (1.15, 2.43)	-0.53 (-2.63, 1.61)	-0.06 (-0.65, 0.53)	2.25 (1.58, 2.92)
25-29 years	-1.30 (-2.25, -0.34)	-2.35 (-2.69, -2.02)	0.98 (0.60, 1.36)	1.40 (0.46, 2.34)	-0.86 (-1.33, -0.38)	1.38 (1.06, 1.69)
30-34 years	-0.83 (-2.36, 0.73)	-2.09 (-3.00, -1.18)	1.01 (0.30, 1.72)	0.27 (-0.28, 0.83)	-0.35 (-0.86, 0.17)	1.48 (0.92, 2.04)
35-39 years	-0.03 (-1.76, 1.73)	-1.11 (-2.08, -0.13)	1.83 (1.11, 2.56)	-0.06 (-0.87, 0.76)	0.67 (0.09, 1.25)	2.41 (1.67, 3.16)
40-44 years	-1.61 (-3.06, -0.14)	-1.97 (-2.75, -1.18)	1.35 (0.86, 1.84)	-0.08 (-0.69, 0.54)	-0.04 (-0.39, 0.31)	1.50 (1.07, 1.93)
45-49 years	-3.85 (-4.70, -3.00)	-4.31 (-4.79, -3.82)	-0.93 (-1.33, -0.53)	-0.48 (-1.44, 0.48)	-2.10 (-2.54, -1.66)	-0.78 (-1.20, -0.35)

CI: confidence interval; EAPC: estimated annual percentage change; NA, not available. [a]Gallbladder and biliary tract cancers.

Supplementary Table S14: Age-period-cohort analysis of incidences of early-onset gastrointestinal cancers in males

	Age-period-cohort analysis for incidence					
**Males**	**Esophageal cancer**	**Gastric cancer**	**Colorectal cancer**	**Liver cancer**	**Gallbladder cancer[a]**	**Pancreatic cancer**
Local drifts[b]						
15-19 years	NA	-0.94 (-3.16, 1.34)	1.05 (-0.47, 2.59)	-2.39 (-4.20, -0.56)	NA	0.19 (-1.51, 1.92)
20-24 years	-1.60 (-3.27, 0.09)	-0.11 (-1.22, 1.01)	2.17 (1.27, 3.07)	-1.67 (-2.63, -0.69)	3.02 (1.61, 4.45)	1.15 (0.21, 2.10)
25-29 years	-2.50 (-3.46, -1.54)	-0.43 (-1.06, 0.21)	2.45 (1.88, 3.03)	-1.20 (-1.69, -0.70)	3.03 (2.27, 3.80)	1.20 (0.65, 1.75)
30-34 years	-3.05 (-3.59, -2.49)	-0.95 (-1.36, -0.55)	2.55 (2.13, 2.97)	-1.09 (-1.40, -0.78)	2.85 (2.38, 3.33)	1.00 (0.67, 1.33)
35-39 years	-3.52 (-3.89, -3.16)	-1.65 (-1.96, -1.34)	2.42 (2.07, 2.77)	-0.88 (-1.12, -0.64)	2.27 (1.92, 2.62)	0.60 (0.37, 0.84)
40-44 years	-3.42 (-3.65, -3.19)	-2.01 (-2.25, -1.78)	2.37 (2.07, 2.67)	-0.66 (-0.85, -0.47)	1.83 (1.56, 2.10)	0.52 (0.34, 0.70)
45-49 years	-2.76 (-2.97, -2.54)	-2.08 (-2.33, -1.83)	2.43 (2.07, 2.80)	-0.09 (-0.31, 0.14)	1.36 (1.04, 1.68)	0.66 (0.45, 0.86)
Age effects[c]						
15-19 years	NA	0.63 (0.49, 0.81)	0.48 (0.39, 0.59)	0.47 (0.39, 0.57)	NA	0.04 (0.04, 0.05)
20-24 years	0.50 (0.41, 0.61)	1.51 (1.28, 1.77)	0.90 (0.77, 1.05)	0.92 (0.80, 1.05)	0.02 (0.02, 0.03)	0.09 (0.08, 0.10)
25-29 years	0.76 (0.66, 0.88)	3.35 (3.01, 3.73)	2.06 (1.84, 2.30)	2.55 (2.36, 2.77)	0.06 (0.05, 0.06)	0.21 (0.19, 0.23)
30-34 years	2.07 (1.91, 2.26)	8.64 (8.06, 9.26)	5.06 (4.66, 5.49)	6.28 (5.95, 6.62)	0.20 (0.18, 0.21)	0.74 (0.69, 0.78)
35-39 years	5.53 (5.23, 5.85)	16.67 (15.79, 17.60)	9.71 (9.06, 10.40)	11.52 (11.03, 12.04)	0.50 (0.47, 0.53)	2.02 (1.94, 2.11)
40-44 years	13.60 (13.00, 14.23)	29.54 (28.17, 30.97)	17.46 (16.43, 18.56)	19.05 (18.30, 19.82)	1.11 (1.05, 1.17)	3.91 (3.77, 4.06)
45-49 years	23.79 (22.79, 24.83)	44.45 (42.49, 46.51)	29.63 (28.02, 31.33)	27.83 (26.79, 28.90)	1.63 (1.55, 1.72)	6.12 (5.92, 6.34)
Period effects[d]						
1992-1996	1.00	1.00	1.00	1.00	1.00	1.00
1997-2001	0.85 (0.80, 0.90)	0.95 (0.89, 1.02)	0.98 (0.90, 1.07)	0.97 (0.92, 1.02)	1.03 (0.95, 1.11)	0.98 (0.93, 1.04)
2002-2006	0.73 (0.68, 0.79)	0.94 (0.87, 1.02)	1.05 (0.96, 1.15)	0.76 (0.71, 0.81)	1.20 (1.10, 1.31)	0.99 (0.93, 1.05)
2007-2011	0.59 (0.53, 0.64)	0.86 (0.78, 0.94)	1.20 (1.10, 1.32)	0.71 (0.66, 0.77)	1.41 (1.28, 1.55)	1.04 (0.97, 1.12)
2012-2016	0.50 (0.45, 0.56)	0.78 (0.70, 0.87)	1.40 (1.27, 1.54)	0.75 (0.69, 0.82)	1.58 (1.44, 1.74)	1.11 (1.03, 1.21)
2017-2021	0.53 (0.47, 0.59)	0.76 (0.68, 0.86)	1.70 (1.54, 1.87)	0.79 (0.72, 0.87)	1.71 (1.55, 1.88)	1.20 (1.10, 1.32)
Birth cohort effects[d]						
1945-1949	1.35 (1.25, 1.46)	1.38 (1.27, 1.50)	0.68 (0.60, 0.77)	0.94 (0.87, 1.01)	0.84 (0.76, 0.94)	0.86 (0.80, 0.92)
1950-1954	1.28 (1.20, 1.35)	1.25 (1.17, 1.33)	0.76 (0.70, 0.84)	1.02 (0.97, 1.08)	0.86 (0.79, 0.93)	0.89 (0.85, 0.94)
1955-1959	1.16 (1.10, 1.22)	1.12 (1.06, 1.19)	0.88 (0.81, 0.95)	0.97 (0.92, 1.02)	0.97 (0.90, 1.03)	0.97 (0.93, 1.01)
1960-1964	1.00	1.00	1.00	1.00	1.00	1.00
1965-1969	0.89 (0.84, 0.93)	0.95 (0.90, 1.00)	1.16 (1.08, 1.23)	1.02 (0.98, 1.07)	1.09 (1.02, 1.15)	1.03 (0.99, 1.07)
1970-1974	0.65 (0.61, 0.69)	0.80 (0.75, 0.84)	1.19 (1.12, 1.28)	0.90 (0.86, 0.95)	1.17 (1.10, 1.24)	0.98 (0.94, 1.02)
1975-1979	0.55 (0.50, 0.59)	0.76 (0.71, 0.82)	1.40 (1.29, 1.52)	0.84 (0.80, 0.89)	1.42 (1.32, 1.53)	1.06 (1.00, 1.12)
1980-1984	0.50 (0.45, 0.57)	0.77 (0.70, 0.84)	1.64 (1.49, 1.81)	0.81 (0.75, 0.87)	1.69 (1.53, 1.87)	1.17 (1.09, 1.25)
1985-1989	0.49 (0.41, 0.59)	0.82 (0.73, 0.93)	1.89 (1.68, 2.13)	0.80 (0.72, 0.87)	1.97 (1.73, 2.26)	1.30 (1.18, 1.43)
1990-1994	0.44 (0.32, 0.60)	0.80 (0.66, 0.98)	1.98 (1.66, 2.36)	0.73 (0.62, 0.86)	2.18 (1.72, 2.75)	1.30 (1.09, 1.54)
1995-1999	0.42 (0.24, 0.74)	0.73 (0.51, 1.05)	1.99 (1.50, 2.64)	0.55 (0.40, 0.76)	2.48 (1.59, 3.88)	1.27 (0.94, 1.71)
2000-2004	NA	0.57 (0.27, 1.20)	1.78 (1.09, 2.90)	0.46 (0.25, 0.85)	NA	1.08 (0.62, 1.88)

NA, not available. [a]Gallbladder and biliary tract cancers. [b]Local drifts indicate the annual percentage change of incidence (% per year for each age group, along with their corresponding 95% confidence intervals. [c]Expected age-specific rates (per 100,000) and 95% confidence intervals adjusted for period effects. [d]Relative risk with 95% confidence intervals.

Supplementary Table S15: Age-period-cohort analysis of incidences of early-onset gastrointestinal cancers in females

	Age-period-cohort analysis for incidence					
**Females**	**Esophageal cancer**	**Gastric cancer**	**Colorectal cancer**	**Liver cancer**	**Gallbladder cancer**[a]	**Pancreatic cancer**
Local drifts[b]						
15-19 years	NA	-1.67 (-3.10, -0.21)	-0.35 (-1.34, 0.66)	-3.01 (-4.33, -1.67)	NA	-1.46 (-2.39, -0.51)
20-24 years	-2.21 (-3.24, -1.16)	-1.13 (-1.83, -0.43)	0.22 (-0.35, 0.80)	-2.97 (-3.70, -2.23)	0.45 (-0.31, 1.21)	-0.88 (-1.39, -0.37)
25-29 years	-2.62 (-3.23, -2.01)	-1.37 (-1.77, -0.98)	0.33 (-0.03, 0.70)	-2.86 (-3.29, -2.42)	0.33 (-0.10, 0.77)	-0.75 (-1.05, -0.44)
30-34 years	-3.23 (-3.63, -2.82)	-1.88 (-2.15, -1.61)	0.19 (-0.07, 0.45)	-2.65 (-2.96, -2.35)	0.11 (-0.18, 0.41)	-0.83 (-1.04, -0.63)
35-39 years	-4.03 (-4.32, -3.74)	-2.54 (-2.76, -2.32)	0.03 (-0.18, 0.25)	-2.42 (-2.67, -2.17)	-0.12 (-0.34, 0.11)	-0.94 (-1.09, -0.78)
40-44 years	-4.77 (-4.96, -4.57)	-3.03 (-3.22, -2.85)	0.11 (-0.08, 0.29)	-2.14 (-2.34, -1.93)	-0.20 (-0.36, -0.03)	-0.85 (-0.96, -0.73)
45-49 years	-5.09 (-5.27, -4.92)	-3.30 (-3.52, -3.09)	0.36 (0.13, 0.59)	-1.68 (-1.90, -1.47)	-0.30 (-0.47, -0.12)	-0.63 (-0.75, -0.50)
Age effects[c]						
15-19 years	NA	0.58 (0.49, 0.67)	0.54 (0.48, 0.61)	0.28 (0.24, 0.32)	NA	0.04 (0.04, 0.05)
20-24 years	0.21 (0.19, 0.24)	1.41 (1.28, 1.55)	0.96 (0.88, 1.06)	0.54 (0.49, 0.59)	0.04 (0.04, 0.04)	0.09 (0.08, 0.09)
25-29 years	0.26 (0.24, 0.29)	3.04 (2.85, 3.25)	1.85 (1.73, 1.99)	0.91 (0.85, 0.97)	0.07 (0.06, 0.08)	0.17 (0.16, 0.17)
30-34 years	0.45 (0.42, 0.48)	5.44 (5.18, 5.70)	3.79 (3.60, 3.98)	1.43 (1.35, 1.50)	0.14 (0.13, 0.14)	0.34 (0.33, 0.35)
35-39 years	0.97 (0.93, 1.02)	8.45 (8.11, 8.80)	6.54 (6.27, 6.82)	2.10 (2.01, 2.20)	0.34 (0.33, 0.36)	0.74 (0.72, 0.76)
40-44 years	2.16 (2.08, 2.24)	11.92 (11.49, 12.37)	10.75 (10.35, 11.18)	3.25 (3.11, 3.39)	0.67 (0.65, 0.70)	1.50 (1.47, 1.54)
45-49 years	3.45 (3.33, 3.58)	13.48 (12.98, 14.00)	15.35 (14.80, 15.93)	5.31 (5.10, 5.52)	1.22 (1.18, 1.26)	2.44 (2.39, 2.50)
Period effects[d]						
1992-1996	1.00	1.00	1.00	1.00	1.00	1.00
1997-2001	0.96 (0.92, 1.01)	0.94 (0.89, 0.98)	0.93 (0.88, 0.97)	0.92 (0.87, 0.96)	0.94 (0.89, 0.98)	0.93 (0.90, 0.96)
2002-2006	0.88 (0.83, 0.93)	0.88 (0.84, 0.93)	0.86 (0.81, 0.90)	0.65 (0.62, 0.69)	0.95 (0.91, 1.00)	0.83 (0.80, 0.86)
2007-2011	0.66 (0.62, 0.71)	0.77 (0.72, 0.82)	0.89 (0.84, 0.94)	0.57 (0.54, 0.61)	0.98 (0.93, 1.03)	0.81 (0.78, 0.84)
2012-2016	0.48 (0.44, 0.51)	0.65 (0.60, 0.70)	0.93 (0.87, 0.98)	0.55 (0.51, 0.59)	0.96 (0.91, 1.02)	0.79 (0.75, 0.82)
2017-2021	0.44 (0.40, 0.47)	0.60 (0.56, 0.65)	1.04 (0.98, 1.11)	0.56 (0.52, 0.61)	1.00 (0.94, 1.06)	0.81 (0.77, 0.86)
Birth cohort effects[d]						
1945-1949	2.35 (2.21, 2.50)	1.73 (1.61, 1.86)	0.89 (0.82, 0.96)	1.25 (1.16, 1.35)	1.03 (0.97, 1.10)	1.03 (0.98, 1.07)
1950-1954	1.84 (1.75, 1.93)	1.44 (1.37, 1.51)	0.95 (0.90, 1.00)	1.20 (1.13, 1.27)	1.01 (0.96, 1.06)	1.02 (0.99, 1.05)
1955-1959	1.35 (1.30, 1.41)	1.20 (1.15, 1.26)	1.00 (0.96, 1.05)	1.05 (1.00, 1.11)	1.03 (0.99, 1.07)	1.04 (1.01, 1.07)
1960-1964	1.00	1.00	1.00	1.00	1.00	1.00
1965-1969	0.82 (0.79, 0.86)	0.88 (0.85, 0.92)	1.00 (0.95, 1.04)	0.96 (0.92, 1.01)	0.98 (0.95, 1.02)	0.94 (0.91, 0.96)
1970-1974	0.65 (0.62, 0.68)	0.74 (0.71, 0.78)	0.98 (0.93, 1.02)	0.80 (0.76, 0.84)	0.95 (0.91, 0.99)	0.87 (0.85, 0.90)
1975-1979	0.54 (0.50, 0.57)	0.67 (0.63, 0.71)	1.00 (0.95, 1.05)	0.67 (0.63, 0.71)	0.99 (0.94, 1.04)	0.85 (0.82, 0.88)
1980-1984	0.49 (0.44, 0.54)	0.64 (0.60, 0.69)	1.02 (0.96, 1.09)	0.59 (0.55, 0.64)	1.00 (0.94, 1.07)	0.83 (0.80, 0.87)
1985-1989	0.45 (0.40, 0.51)	0.64 (0.59, 0.69)	1.05 (0.97, 1.13)	0.54 (0.49, 0.59)	1.02 (0.93, 1.11)	0.80 (0.76, 0.85)
1990-1994	0.41 (0.34, 0.50)	0.60 (0.53, 0.68)	1.07 (0.95, 1.19)	0.45 (0.39, 0.52)	1.06 (0.92, 1.21)	0.76 (0.69, 0.84)
1995-1999	0.35 (0.25, 0.50)	0.53 (0.42, 0.67)	1.01 (0.84, 1.21)	0.36 (0.28, 0.46)	1.07 (0.84, 1.36)	0.69 (0.58, 0.81)
2000-2004	NA	0.42 (0.26, 0.69)	0.89 (0.64, 1.23)	0.32 (0.21, 0.50)	NA	0.58 (0.42, 0.79)

NA, not available. [a]Gallbladder and biliary tract cancers. [b]Local drifts indicate the annual percentage change of incidence (% per year) for each age group, along with their corresponding 95% confidence intervals. [c]Expected age-specific rates (per 100,000) and 95% confidence intervals adjusted for period effects. [d]Relative risk with 95% confidence intervals.

Supplementary Table S16: Age-period-cohort analysis of mortality for early-onset gastrointestinal cancers in males

	Age-period-cohort analysis for mortality					
**Males**	**Esophageal cancer**	**Gastric cancer**	**Colorectal cancer**	**Liver cancer**	**Gallbladder cancer[a]**	**Pancreatic cancer**
Local drifts[b]						
15-19 years	NA	-3.22 (-5.52, -0.87)	-1.76 (-3.33, -0.17)	-3.07 (-5.16, -0.94)	NA	-0.06 (-1.72, 1.63)
20-24 years	-2.46 (-4.10, -0.78)	-2.37 (-3.49, -1.23)	-0.71 (-1.61, -0.19)	-2.33 (-3.43, -1.21)	0.42 (-0.69, 1.55)	0.89 (-0.03, 1.80)
25-29 years	-3.35 (-4.31, -2.38)	-2.51 (-3.15, -1.87)	-0.38 (-0.94, 0.19)	-1.91 (-2.48, -1.33)	0.43 (-0.17, 1.03)	0.92 (0.38, 1.46)
30-34 years	-3.88 (-4.43, -3.31)	-2.75 (-3.14, -2.37)	-0.15 (-0.55, 0.25)	-1.74 (-2.11, -1.38)	0.33 (-0.03, 0.69)	0.75 (0.42, 1.08)
35-39 years	-4.34 (-4.72, -3.96)	-3.17 (-3.46, -2.89)	-0.18 (-0.51, 0.15)	-1.61 (-1.90, -1.33)	-0.08 (-0.35, 0.18)	0.37 (0.13, 0.61)
40-44 years	-4.23 (-4.47, -4.00)	-3.36 (-3.56, -3.15)	-0.20 (-0.48, 0.08)	-1.47 (-1.69, -1.24)	-0.29 (-0.49, -0.10)	0.32 (0.14, 0.50)
45-49 years	-3.60 (-3.81, -3.39)	-3.33 (-3.54, -3.12)	-0.18 (-0.50, 0.14)	-0.99 (-1.25, -0.73)	-0.46 (-0.68, -0.24)	0.47 (0.27, 0.68)
Age effects[c]						
15-19 years	NA	0.58 (0.46, 0.73)	0.49 (0.41, 0.59)	0.51 (0.41, 0.64)	NA	0.05 (0.04, 0.06)
20-24 years	0.54 (0.45, 0.66)	1.27 (1.10, 1.46)	0.81 (0.71, 0.93)	0.99 (0.86, 1.14)	0.03 (0.02, 0.03)	0.09 (0.08, 0.11)
25-29 years	0.72 (0.63, 0.83)	2.28 (2.06, 2.51)	1.45 (1.30, 1.61)	2.41 (2.19, 2.64)	0.06 (0.05, 0.06)	0.20 (0.18, 0.22)
30-34 years	1.86 (1.72, 2.03)	5.76 (5.41, 6.12)	3.06 (2.84, 3.30)	5.61 (5.27, 5.96)	0.17 (0.16, 0.18)	0.67 (0.64, 0.72)
35-39 years	4.52 (4.28, 4.78)	11.32 (10.81, 11.86)	5.32 (4.99, 5.66)	9.49 (9.01, 9.98)	0.36 (0.35, 0.38)	1.74 (1.67, 1.82)
40-44 years	11.11 (10.63, 11.62)	20.01 (19.21, 20.83)	8.36 (7.89, 8.85)	15.87 (15.16, 16.62)	0.74 (0.71, 0.77)	3.47 (3.35, 3.60)
45-49 years	18.95 (18.14, 19.78)	29.51 (28.38, 30.69)	12.84 (12.16, 13.55)	21.99 (21.03, 23.00)	1.13 (1.09, 1.18)	5.58 (5.39, 5.78)
Period effects[d]						
1992-1996	1.00	1.00	1.00	1.00	1.00	1.00
1997-2001	0.84 (0.79, 0.89)	0.89 (0.84, 0.94)	0.90 (0.84, 0.97)	0.98 (0.92, 1.04)	0.95 (0.90, 1.01)	0.97 (0.92, 1.03)
2002-2006	0.70 (0.65, 0.76)	0.80 (0.75, 0.86)	0.85 (0.79, 0.92)	0.74 (0.69, 0.80)	1.01 (0.95, 1.07)	0.97 (0.91, 1.03)
2007-2011	0.53 (0.48, 0.58)	0.65 (0.59, 0.71)	0.82 (0.75, 0.89)	0.67 (0.62, 0.73)	1.02 (0.96, 1.10)	1.01 (0.94, 1.08)
2012-2016	0.43 (0.39, 0.48)	0.54 (0.49, 0.60)	0.83 (0.76, 0.92)	0.65 (0.59, 0.72)	1.01 (0.94, 1.09)	1.06 (0.98, 1.16)
2017-2021	0.43 (0.38, 0.48)	0.49 (0.44, 0.55)	0.89 (0.81, 0.98)	0.67 (0.60, 0.75)	0.98 (0.91, 1.06)	1.14 (1.04, 1.24)
Birth cohort effects[d]						
1945-1949	1.55 (1.44, 1.68)	1.66 (1.54, 1.78)	1.02 (0.91, 1.13)	1.12 (1.03, 1.22)	1.07 (0.99, 1.15)	0.88 (0.82, 0.94)
1950-1954	1.40 (1.32, 1.48)	1.41 (1.34, 1.49)	1.00 (0.93, 1.09)	1.13 (1.06, 1.21)	1.03 (0.97, 1.09)	0.91 (0.86, 0.96)
1955-1959	1.21 (1.15, 1.28)	1.19 (1.14, 1.25)	1.01 (0.94, 1.08)	1.03 (0.97, 1.09)	1.06 (1.01, 1.12)	0.98 (0.94, 1.02)
1960-1964	1.00	1.00	1.00	1.00	1.00	1.00
1965-1969	0.85 (0.81, 0.90)	0.89 (0.85, 0.93)	1.03 (0.96, 1.09)	1.00 (0.95, 1.06)	0.98 (0.94, 1.03)	1.02 (0.98, 1.06)
1970-1974	0.60 (0.57, 0.64)	0.69 (0.66, 0.73)	0.94 (0.88, 1.01)	0.85 (0.81, 0.90)	0.95 (0.90, 0.99)	0.96 (0.92, 1.00)
1975-1979	0.48 (0.45, 0.53)	0.61 (0.57, 0.65)	0.97 (0.89, 1.05)	0.75 (0.70, 0.81)	1.00 (0.94, 1.06)	1.02 (0.97, 1.08)
1980-1984	0.43 (0.38, 0.48)	0.55 (0.50, 0.60)	0.98 (0.89, 1.08)	0.71 (0.65, 0.78)	1.04 (0.96, 1.13)	1.11 (1.04, 1.20)
1985-1989	0.40 (0.33, 0.48)	0.52 (0.46, 0.58)	0.97 (0.86, 1.09)	0.68 (0.61, 0.76)	1.07 (0.96, 1.19)	1.22 (1.10, 1.34)
1990-1994	0.34 (0.25, 0.47)	0.45 (0.36, 0.55)	0.88 (0.74, 1.06)	0.59 (0.49, 0.72)	1.05 (0.87, 1.27)	1.20 (1.01, 1.43)
1995-1999	0.32 (0.18, 0.55)	0.36 (0.25, 0.53)	0.78 (0.58, 1.04)	0.44 (0.30, 0.63)	1.06 (0.74, 1.52)	1.17 (0.87, 1.56)
2000-2004	NA	0.25 (0.11, 0.56)	0.61 (0.36, 1.03)	0.35 (0.17, 0.71)	NA	0.98 (0.57, 1.69)

NA, not available. [a]Gallbladder and biliary tract cancers. [b]Local drifts indicate the annual percentage change of mortality (% per year) for each age group, along with their corresponding 95% confidence intervals. [c]Expected age-specific rates (per 100,000) and 95% confidence intervals adjusted for period effects. [d]Relative risk with 95% confidence intervals.

Supplementary Table S17: Age-period-cohort analysis of mortality for early-onset gastrointestinal cancers in females

	Age-period-cohort analysis for mortality					
**Females**	**Esophageal cancer**	**Gastric cancer**	**Colorectal cancer**	**Liver cancer**	**Gallbladder cancer[a]**	**Pancreatic cancer**
Local drifts[b]						
15-19 years	NA	-3.84 (-5.11, -2.55)	-3.32 (-4.32, -2.31)	-3.77 (-5.29, -2.23)	NA	-1.78 (-2.76, -0.79)
20-24 years	-3.84 (-4.90, -2.77)	-3.27 (-3.87, -2.66)	-2.77 (-3.33, -2.21)	-3.75 (-4.58, -2.91)	-1.72 (-2.66, -0.77)	-1.21 (-1.73, -0.69)
25-29 years	-4.23 (-4.85, -3.60)	-3.38 (-3.72, -3.04)	-2.66 (-3.00, -2.31)	-3.70 (-4.20, -3.20)	-1.87 (-2.41, -1.32)	-1.09 (-1.40, -0.77)
30-34 years	-4.82 (-5.23, -4.40)	-3.65 (-3.87, -3.42)	-2.69 (-2.93, -2.44)	-3.45 (-3.80, -3.10)	-2.07 (-2.44, -1.71)	-1.16 (-1.38, -0.95)
35-39 years	-5.60 (-5.90, -5.30)	-4.05 (-4.22, -3.87)	-2.72 (-2.92, -2.52)	-3.20 (-3.49, -2.90)	-2.20 (-2.47, -1.92)	-1.24 (-1.41, -1.08)
40-44 years	-6.33 (-6.52, -6.13)	-4.34 (-4.49, -4.20)	-2.61 (-2.78, -2.45)	-2.89 (-3.12, -2.65)	-2.11 (-2.31, -1.91)	-1.12 (-1.24, -1.00)
45-49 years	-6.66 (-6.84, -6.49)	-4.50 (-4.65, -4.34)	-2.40 (-2.60, -2.21)	-2.40 (-2.65, -2.15)	-1.95 (-2.14, -1.75)	-0.85 (-0.98, -0.72)
Age effects[c]						
15-19 years	NA	0.55 (0.49, 0.62)	0.54 (0.49, 0.60)	0.31 (0.26, 0.36)	NA	0.04 (0.04, 0.05)
20-24 years	0.23 (0.21, 0.26)	1.24 (1.15, 1.33)	0.85 (0.78, 0.92)	0.58 (0.52, 0.64)	0.05 (0.04, 0.05)	0.09 (0.08, 0.10)
25-29 years	0.24 (0.22, 0.26)	2.18 (2.07, 2.29)	1.26 (1.19, 1.34)	0.85 (0.79, 0.93)	0.07 (0.06, 0.07)	0.16 (0.15, 0.16)
30-34 years	0.37 (0.34, 0.39)	3.82 (3.68, 3.96)	2.20 (2.10, 2.29)	1.27 (1.19, 1.35)	0.12 (0.11, 0.13)	0.31 (0.30, 0.32)
35-39 years	0.69 (0.66, 0.73)	5.93 (5.75, 6.11)	3.43 (3.31, 3.56)	1.72 (1.63, 1.81)	0.26 (0.24, 0.27)	0.63 (0.61, 0.65)
40-44 years	1.48 (1.43, 1.54)	8.31 (8.09, 8.55)	4.90 (4.73, 5.07)	2.71 (2.58, 2.85)	0.47 (0.45, 0.49)	1.31 (1.28, 1.34)
45-49 years	2.22 (2.14, 2.30)	9.26 (9.00, 9.53)	6.22 (6.01, 6.43)	4.31 (4.11, 4.51)	0.87 (0.84, 0.91)	2.21 (2.16, 2.26)
Period effects[d]						
1992-1996	1.00	1.00	1.00	1.00	1.00	1.00
1997-2001	0.93 (0.89, 0.97)	0.87 (0.85, 0.90)	0.85 (0.81, 0.88)	0.92 (0.87, 0.98)	0.87 (0.83, 0.92)	0.92 (0.89, 0.95)
2002-2006	0.79 (0.75, 0.84)	0.76 (0.73, 0.79)	0.68 (0.65, 0.71)	0.62 (0.58, 0.67)	0.82 (0.77, 0.87)	0.81 (0.78, 0.84)
2007-2011	0.53 (0.50, 0.57)	0.58 (0.55, 0.61)	0.58 (0.55, 0.61)	0.52 (0.49, 0.57)	0.73 (0.69, 0.78)	0.77 (0.74, 0.81)
2012-2016	0.35 (0.32, 0.38)	0.45 (0.42, 0.48)	0.52 (0.50, 0.56)	0.47 (0.43, 0.51)	0.64 (0.60, 0.69)	0.74 (0.70, 0.78)
2017-2021	0.29 (0.27, 0.32)	0.40 (0.37, 0.42)	0.52 (0.49, 0.55)	0.47 (0.43, 0.52)	0.61 (0.57, 0.65)	0.76 (0.71, 0.80)
Birth cohort effects[d]						
1945-1949	3.04 (2.86, 3.23)	2.06 (1.96, 2.17)	1.38 (1.29, 1.47)	1.42 (1.31, 1.55)	1.28 (1.20, 1.38)	1.05 (1.01, 1.10)
1950-1954	2.18 (2.08, 2.28)	1.62 (1.56, 1.68)	1.26 (1.21, 1.32)	1.29 (1.21, 1.38)	1.19 (1.13, 1.26)	1.04 (1.01, 1.08)
1955-1959	1.48 (1.42, 1.55)	1.28 (1.24, 1.32)	1.16 (1.11, 1.21)	1.10 (1.03, 1.17)	1.13 (1.07, 1.18)	1.05 (1.02, 1.08)
1960-1964	1.00	1.00	1.00	1.00	1.00	1.00
1965-1969	0.76 (0.72, 0.79)	0.83 (0.80, 0.86)	0.88 (0.85, 0.91)	0.95 (0.90, 1.00)	0.90 (0.86, 0.94)	0.93 (0.90, 0.95)
1970-1974	0.56 (0.53, 0.58)	0.65 (0.63, 0.67)	0.75 (0.72, 0.78)	0.75 (0.70, 0.79)	0.78 (0.75, 0.82)	0.85 (0.82, 0.87)
1975-1979	0.42 (0.39, 0.45)	0.54 (0.51, 0.56)	0.66 (0.63, 0.70)	0.59 (0.55, 0.64)	0.72 (0.68, 0.77)	0.81 (0.78, 0.84)
1980-1984	0.35 (0.32, 0.39)	0.46 (0.44, 0.49)	0.58 (0.55, 0.62)	0.51 (0.46, 0.55)	0.65 (0.60, 0.71)	0.78 (0.74, 0.82)
1985-1989	0.30 (0.26, 0.34)	0.40 (0.38, 0.43)	0.51 (0.47, 0.55)	0.45 (0.40, 0.50)	0.59 (0.53, 0.67)	0.74 (0.69, 0.79)
1990-1994	0.25 (0.20, 0.31)	0.34 (0.31, 0.38)	0.45 (0.40, 0.50)	0.36 (0.30, 0.42)	0.56 (0.47, 0.67)	0.69 (0.62, 0.76)
1995-1999	0.20 (0.14, 0.29)	0.27 (0.22, 0.33)	0.37 (0.30, 0.44)	0.27 (0.20, 0.36)	0.51 (0.37, 0.69)	0.62 (0.52, 0.73)
2000-2004	NA	0.19 (0.12, 0.30)	0.28 (0.20, 0.39)	0.23 (0.14, 0.39)	NA	0.51 (0.36, 0.70)

NA, not available. [a]Gallbladder and biliary tract cancers. [b]Local drifts indicate the annual percentage change of mortality (% per year) for each age group, along with their corresponding 95% confidence intervals. [c]Expected age-specific rates (per 100,000) and 95% confidence intervals adjusted for period effects. [d]Relative risk with 95% confidence intervals.
